# Chronic obstructive pulmonary disease and related phenotypes: polygenic risk scores in population-based and case-control cohorts

**DOI:** 10.1016/S2213-2600(20)30101-6

**Published:** 2020-07

**Authors:** Matthew Moll, Phuwanat Sakornsakolpat, Nick Shrine, Brian D Hobbs, Dawn L DeMeo, Catherine John, Anna L Guyatt, Michael J McGeachie, Sina A Gharib, Ma'en Obeidat, Lies Lahousse, Sara R A Wijnant, Guy Brusselle, Deborah A Meyers, Eugene R Bleecker, Xingnan Li, Ruth Tal-Singer, Ani Manichaikul, Stephen S Rich, Sungho Won, Woo Jin Kim, Ah Ra Do, George R Washko, R Graham Barr, Bruce M Psaty, Traci M Bartz, Nadia N Hansel, Kathleen Barnes, John E Hokanson, James D Crapo, David Lynch, Per Bakke, Amund Gulsvik, Ian P Hall, Louise Wain, International COPD Genetics Consortium, SpiroMeta Consortium, María Soler Artigas, Victoria E Jackson, David P Strachan, Jennie Hui, Alan L James, Shona M Kerr, Ozren Polasek, Veronique Vitart, Jonathan Marten, Igor Rudan, Mika Kähönen, Ida Surakka, Christian Gieger, Stefan Karrasch, Rajesh Rawal, Holger Schulz, Ian J Deary, Sarah E Harris, Stefan Enroth, Ulf Gyllensten, Medea Imboden, Nicole M Probst-Hensch, Terho Lehtimäki, Olli T Raitakari, Claudia Langenberg, Jian'an Luan, Nick Wareham, Jing Hua Zhao, Caroline Hayward, Alison Murray, David J Porteous, Blair H Smith, Marjo-Riitta Jarvelin, Matthias Wielscher, Peter K Joshi, Katherine A Kentistou, Paul RHJ Timmers, James F Wilson, James P Cook, Lars Lind, Anubha Mahajan, Andrew P Morris, Ralf Ewert, Georg Homuth, Beate Stubbe, Stefan Weiss, Eleftheria Zeggini, Scott T Weiss, Edwin K Silverman, Frank Dudbridge, Martin D Tobin, Michael H Cho

**Affiliations:** a Channing Division of Network Medicine, Brigham and Women's Hospital, Boston, MA, USA; b Division of Pulmonary and Critical Care Medicine, Brigham and Women's Hospital, Boston, MA, USA; c Department of Medicine, Faculty of Medicine Siriraj Hospital, Mahidol University, Bangkok, Thailand; d Genetic Epidemiology Group, Department of Health Sciences, University of Leicester, Leicester, UK; e Computational Medicine Core, Center for Lung Biology, Department of Medicine, University of Washington, Seattle, WA, USA; f Division of Pulmonary, Critical Care, and Sleep Medicine, Department of Medicine, University of Washington, Seattle, WA, USA; g Cardiovascular Health Research Unit, Department of Medicine, University of Washington, Seattle, WA, USA; h University of British Columbia Center for Heart Lung Innovation, St Paul's Hospital, Vancouver, BC, Canada; i Department of Epidemiology, Erasmus Medical Centre, Rotterdam, Netherlands; j Department of Respiratory Medicine, Erasmus Medical Centre, Rotterdam, Netherlands; k Department of Bioanalysis, Faculty of Pharmaceutical Sciences, Ghent University, Ghent, Belgium; l Department of Respiratory Medicine, Ghent University Hospital, Ghent, Belgium; m Department of Medicine, University of Arizona, Tucson, AZ, USA; n GlaxoSmithKline Research and Development, Collegeville, PA, USA; o Center for Public Health Genomics, University of Virginia, Charlottesville, VA, USA; p Department of Public Health Sciences, University of Virginia, Charlottesville, VA, USA; q Department of Public Health Sciences, Graduate School of Public Health, Seoul National University, Seoul, South Korea; r Interdisciplinary Program of Bioinformatics, College of National Sciences, Seoul National University, Seoul, South Korea; s Institute of Health and Environment, Seoul National University, Seoul, South Korea; t Department of Internal Medicine, Kangwon National University, Chuncheon, South Korea; u Department of Medicine and Department of Epidemiology, Columbia University Medical Center, New York, NY, USA; v Kaiser Permanente Washington Health Research Institute, Seattle, WA; w School of Medicine, Johns Hopkins University, Baltimore, MD, USA; x Colorado Center for Personalized Medicine, University of Colorado Anschutz Medical Campus, Aurora, CO, USA; y School of Public Health, University of Colorado, Denver; z Division of Pulmonary, Critical Care, and Sleep Medicine, National Jewish Health, Denver, CO, USA; aa Department of Radiology, National Jewish Health, Denver, CO, USA; ab Department of Clinical Science, University of Bergen, Bergen, Norway; ac Division of Respiratory Medicine, Queen's Medical Centre, Nottingham, UK; ad National Institute for Health Research Leicester Respiratory Biomedical Research Centre, Glenfield Hospital, Leicester, UK; ae Harvard Medical School, Boston, MA, USA

## Abstract

**Background:**

Genetic factors influence chronic obstructive pulmonary disease (COPD) risk, but the individual variants that have been identified have small effects. We hypothesised that a polygenic risk score using additional variants would predict COPD and associated phenotypes.

**Methods:**

We constructed a polygenic risk score using a genome-wide association study of lung function (FEV_1_ and FEV_1_/forced vital capacity [FVC]) from the UK Biobank and SpiroMeta. We tested this polygenic risk score in nine cohorts of multiple ethnicities for an association with moderate-to-severe COPD (defined as FEV_1_/FVC <0·7 and FEV_1_ <80% of predicted). Associations were tested using logistic regression models, adjusting for age, sex, height, smoking pack-years, and principal components of genetic ancestry. We assessed predictive performance of models by area under the curve. In a subset of studies, we also studied quantitative and qualitative CT imaging phenotypes that reflect parenchymal and airway pathology, and patterns of reduced lung growth.

**Findings:**

The polygenic risk score was associated with COPD in European (odds ratio [OR] per SD 1·81 [95% CI 1·74–1·88] and non-European (1·42 [1·34–1·51]) populations. Compared with the first decile, the tenth decile of the polygenic risk score was associated with COPD, with an OR of 7·99 (6·56–9·72) in European ancestry and 4·83 (3·45–6·77) in non-European ancestry cohorts. The polygenic risk score was superior to previously described genetic risk scores and, when combined with clinical risk factors (ie, age, sex, and smoking pack-years), showed improved prediction for COPD compared with a model comprising clinical risk factors alone (AUC 0·80 [0·79–0·81] *vs* 0·76 [0·75–0·76]). The polygenic risk score was associated with CT imaging phenotypes, including wall area percent, quantitative and qualitative measures of emphysema, local histogram emphysema patterns, and destructive emphysema subtypes. The polygenic risk score was associated with a reduced lung growth pattern.

**Interpretation:**

A risk score comprised of genetic variants can identify a small subset of individuals at markedly increased risk for moderate-to-severe COPD, emphysema subtypes associated with cigarette smoking, and patterns of reduced lung growth.

**Funding:**

US National Institutes of Health, Wellcome Trust.

## Introduction

Chronic obstructive pulmonary disease (COPD) is characterised by irreversible airflow limitation. The Global Initiative for Chronic Obstructive Lung Disease (GOLD) defines COPD by the presence of persistent respiratory symptoms with airflow obstruction based on a low FEV_1_/forced vital capacity (FVC) ratio, and grades spirometric severity on the basis of decrements in post-bronchodilator percentage of predicted FEV_1_ (% predicted).^1^ COPD primarily develops in the context of toxic environmental exposures, including cigarette smoking and biofuel combustion. However, not all exposed individuals develop airflow obstruction,^2^, ^3^ which suggests that some individuals could have a genetic susceptibility to the disease.

Heritability estimates for COPD typically range between 37% and 50%.^4^, ^5^, ^6^ Genome-wide association studies (GWASs) of COPD and lung function have identified numerous genetic variants associated with COPD risk.^7^, ^8^, ^9^, ^10^, ^11^, ^12^, ^13^ The effect size of each of these GWAS variants is generally small. However, although each individual variant only explains a small proportion of COPD risk, the combination of many genetic variants into a single genetic risk score explains a greater proportion of the risk.^7^, ^11^, ^14^, ^15^ Genetic risk scores have been developed for lung function, with predictive power for COPD.^7^, ^13^, ^14^ Genetic risk scores based on larger GWASs, and including more variants, tend to exhibit higher predictive performance.^7^

**Research in context**

**Evidence before this study**

We searched PubMed for studies published up to Sept 28, 2019, using the terms “COPD”, “genetic*”, “risk score”, “COPD”, and “gwa study”, with no language restrictions. Previous research has shown that COPD is influenced by genetic factors, but variants identified by genome-wide association studies (GWASs) are of individually small effect, and account for a modest fraction of genetic risk. Studies combining these variants showed improved risk prediction, but no studies have attempted to include full genome-wide results.

**Added value of this study**

We developed a polygenic risk score using a large GWAS of lung function. This risk score predicted COPD in multiple cohorts, and is associated with a wide range of CT imaging phenotypes and lung growth patterns that are thought to be linked to the development of COPD.

**Implications of all the available evidence**

A polygenic risk score can quantify an individual's risk for COPD independently from, and earlier than, clinical risk factors of age and cigarette smoking. Future research is needed to determine whether these scores can identify individuals most likely to benefit from preventive therapy or targeted trials.

Although genetic risk scores for lung function can predict COPD,^7^, ^11^ the degree to which genetic risk scores can capture COPD heterogeneity is not clear. Individuals with COPD can have widely varying airway and lung parenchymal involvement, and individual COPD GWAS variants are associated with quantitative imaging features, such as airway wall thickness and emphysema.^8^, ^9^, ^10^ Oelsner and colleagues^16^ derived a 79-variant genetic risk score from a previous GWAS for lung function^11^ and identified an association with quantitative imaging features on chest CT. However, whether a genetic risk score comprised of a larger number of lung function variants would result in stronger associations with a wider range of quantitative and qualitative CT imaging features is not known.

Genetic risk scores represent a carefully selected set of variants that are either unweighted, or weighted in the context of other variants in the regression model. We use the term polygenic risk score to refer to risk scores that include variants across the genome, with weights derived from GWASs. In cardiovascular diseases, polygenic risk scores including variants that did not reach genome-wide significance have improved power and identified a large proportion of the population with markedly increased disease risk.^17^ Therefore, it is possible that polygenic risk scores for lung function that include variants not reaching genome-wide significance will be more accurate than genetic risk scores for predicting complex traits such as COPD. In addition, some individuals with reduced lung growth early in life are at risk of developing COPD.^2^, ^18^, ^19^ COPD and lung function GWAS variants are associated with anthropometric features (eg, height) and are enriched in lung development pathways.^7^, ^10^ It is unknown whether risk scores of lung function genetic variants are associated with patterns of lung growth.

We hypothesised that polygenic risk scores developed using the full results of the largest available genome-wide genetic studies of lung function would improve the prediction of COPD and identify individuals at markedly increased risk of disease. Because decreased lung function can occur as a continuum before individuals meet the GOLD spirometry criteria for COPD,^1^ we developed a polygenic risk score based on lung function (ie, FEV_1_ and FEV_1_/FVC ratio) and then tested the predictive power of the polygenic risk score for COPD. We also sought to determine whether the score was related to specific quantitative and qualitative CT imaging phenotypes and patterns of lung growth. To test this hypothesis, we developed individual polygenic risk scores based on FEV_1_ and FEV_1_/FVC ratio, and joined these scores into a combined polygenic risk score. We tested the effect of this combined risk score in nine additional independent cohorts, including both population-based and case-control designs, multiple racial and ethnic groups, and children with asthma.

## Methods

### Study populations

GWASs for FEV_1_ and FEV_1_/FVC were done for participants in the UK Biobank and SpiroMeta.^7^ We used the GenKOLS case-control study from Bergen, Norway^20^, ^21^, ^22^ to tune hyperparameters. We calculated polygenic risk scores in both case-control and population-based studies across a range of ethnicities. Case-control studies included COPDGene (non-Hispanic white and African American participants),^23^ ECLIPSE,^24^ NETT^25^ and Normative Aging Study (NAS),^26^ SPIROMICS,^27^, ^28^ and the Lung Health Study (LHS).^29^, ^30^ Population-based studies included MESA (African American, non-Hispanic white, Hispanic, and Chinese participants),^31^, ^32^ Cardiovascular Health Study (CHS; African American and European ancestry participants),^33^ the Rotterdam Study (all three cohorts),^34^ and a study by Kangwon University.^35^ For lung-function growth patterns, we did an analysis using individuals in the Childhood Asthma Management Program (CAMP).^18^ CAMP was a randomised placebo-controlled trial of anti-inflammatory treatments in 1041 children with mild-to-moderate asthma (aged 5–12 years at enrolment), with 13 years of follow-up and low attrition (≤20%).^36^, ^37^ All participants gave informed consent and study protocols were approved by local Research Ethics Committees and Institutional Review Boards. Additional details of the study populations are available in the appendix (pp 2–5).

### Outcomes

The primary outcome measure was moderate-to-severe COPD (FEV_1_/FVC <0·7 and FEV_1_ <80% of predicted). As secondary outcome measures, we assessed the association of the combined polygenic risk score with smoking, occurrence of exacerbations, GOLD spirometry grades, clinical COPD phenotypes, imaging phenotypes, and lung growth patterns. We also assessed the performance of the polygenic risk scores (alone and in combination with clinical COPD risk factors) in predicting COPD, and compared this with the performance of a clinical risk score.

### Derivation of polygenic risk scores

To develop individual polygenic risk scores for FEV_1_ and FEV_1_/FVC, we generated weights based on effect sizes from GWASs of FEV_1_ and FEV_1_/FVC in the UK Biobank and SpiroMeta.^7^ To reduce the chance of genetic variant drop-out between studies, we included variants that were either genotyped or well imputed (R^2^>0·5) in four cohorts: COPDGene, GenKOLS, ECLIPSE, and NETT/NAS. We then applied a penalised regression framework, accounting for linkage disequilibrium (lassosum v0.4.4),^38^ in which linkage disequilibrium was calculated using European ancestry individuals in the UK Biobank.^39^ To determine hyperparameters (λ and shrinkage) for lassosum, we used the GenKOLS case-control study. We chose GenKOLS to avoid training any model parameters on the COPDGene study to preserve COPDGene for testing, and because GenKOLS was a well powered and balanced case-control study.

The primary outcome measure, moderate-to-severe COPD, requires both reduced FEV_1_ and reduced FEV_1_/FVC for diagnosis according to GOLD criteria. Therefore, we created a single combined polygenic risk score using a weighted sum of the two individual polygenic risk scores for FEV_1_ and FEV_1_/FVC. To achieve this, we built a logistic regression model for COPD that included the individual polygenic risk scores for FEV_1_ and FEV_1_/FVC in GenKOLS, and used their regression coefficients as weights to calculate the combined score.

To test the sensitivity of the scores to the cohort used for parameter tuning, we tuned the polygenic risk scores using one of the four cohorts (COPDGene non-Hispanic white individuals, ECLIPSE, NETT/NAS, or GenKOLS), then tested predictive power in the three other cohorts.

### Statistical analysis

We used the resulting regression model to calculate polygenic risk scores in participants from nine studies (COPDGene, CHS, ECLIPSE, Kangwon University, LHS, MESA, NETT/NAS, Rotterdam Study, and SPIROMICS). For each cohort, FEV_1_ and FEV_1_/FVC polygenic risk scores were centred and scaled by their means and standard deviations.

We checked for correlation of the combined polygenic risk score with smoking pack-years using Pearson correlation coefficients. To estimate the effect of smoking, we calculated the proportion of population-attributable risk (PAR%) and attributable risk in the exposed (AR%) for smoking exposure (dichotomised at 20 pack-years (>20 pack-years *vs* ≤20 pack-years) using COPDGene non-Hispanic white participants.

To assess the primary outcome measure, we tested for association between the polygenic risk score and COPD (moderate-to-severe, unless otherwise stated) using logistic regression models, adjusting for age, sex, height, smoking pack-years, and principal components of genetic ancestry, as well as study clinic in CHS. We tested the polygenic risk score for association with frequent exacerbations (>1 exacerbation in 12 months; exacerbations were defined as self-reported worsening in respiratory health requiring therapy with corticosteroids, antibiotics, or both) and severe exacerbations (exacerbation requiring emergency room visit or hospital admission) in the COPDGene and ECLIPSE cohorts, adjusting for age, sex, and pack-years; these models were then tested again adjusting for baseline FEV_1_ and FEV_1_/FVC. To assess the predictive performance of polygenic risk scores for COPD, we estimated the area under the curve (AUC) using pROC in R version 3.5.1. We evaluated the following models: 1) polygenic risk score alone; 2) traditional COPD clinical risk factors (age, sex, and cigarette smoking pack-years) alone; and 3) COPD clinical risk factors and polygenic risk score. We compared these models in ten subpopulations, resulting in a Bonferroni-corrected p-value of 0.005. We also derived a clinical risk score from UK Biobank participants with 10 or more pack-years of smoking, and estimated AUC in the COPDGene and ECLIPSE studies. Cutoffs for clinical risk score and polygenic risk score were chosen based on the Youden index,^40^ and performance characteristics were calculated. All meta-analyses were performed with the meta package in R (v4.9-7).^41^ Because the polygenic risk scores were developed in European ancestry cohorts, we separately examined European and non-European ancestry cohorts. We performed meta-analyses of AUC by both inverse variance weighting and effective sample size weighting;^42^ these meta-analyses used a fixed-effects approach for Europeans, but we performed a random effects analysis for non-Europeans to account for the diversity of racial ancestry. We grouped participants in each study by combined polygenic risk score deciles and tertiles, and compared highest and lowest deciles to each other and to the middle tertile.

We tested for association between polygenic risk scores and COPD-related phenotypes that were available in some cohorts. We tested for association between polygenic risk scores for FEV_1_ and GOLD spirometry grades in the COPDGene study, which includes well characterised categories of heavy smokers who are at risk for COPD but do not meet spirometric criteria for the disease, including participants with normal spirometry and preserved ratio with impaired spirometry (PRISm). We also tested the association of polygenic risk scores with quantitative imaging phenotypes: quantitative emphysema on inspiratory CT scans (% low attenuation area [LAA] of less than –950 Hounsfield units [HU]),^43^ mean wall area percent (WAP),^43^ 15th percentile of the lung density histogram on inspiratory CT scans (Perc15),^44^ square root of wall area of a hypothetical airway with an internal perimeter of 10 mm (Pi10),^45^ and gas trapping on expiratory CT (less than –856 HU).^46^ When testing for association with imaging phenotypes, we fitted linear regression models adjusted for age, sex, smoking pack-years, CT scanner type, height (for Pi10 and WAP), study centre (gas trapping only) and principal components of genetic ancestry. % LAA less than –950 HU and gas trapping were log-transformed before analyses. We also tested the association of polygenic risk scores with qualitative imaging phenotypes: qualitative emphysema,^47^ local histogram patterns of emphysema^48^, ten CT subtypes that were defined in COPDGene,^49^ and visual emphysema severity on the basis of Fleischner guidelines.^50^ Local histogram patterns of emphysema were log-transformed before analyses. Associations of polygenic risk scores with local histogram patterns of emphysema were tested with Tobit regression using the VGAM R package.^51^ We tested for association with visual emphysema severity with ordinal logistic regression using the MASS R package.^52^ For imaging phenotypes, we considered a total of 20 phenotypes (Pi10, WAP, Perc15, %LAA less than –950 HU, gas trapping, qualitative emphysema, five local histogram phenotypes, and nine subtypes), resulting in a Bonferroni-corrected p value of 0·0025.

For the lung-function growth pattern analysis, we applied logistic regression to compare reduced lung growth patterns to normal growth patterns, and we performed pairwise comparisons combining patterns of normal lung growth (normal growth with normal decline, and normal growth with early decline) and reduced lung growth (reduced growth with normal decline, and reduced growth with early decline). We adjusted for age, sex, height, baseline FEV_1_, percentage bronchodilator response (change from baseline FEV_1_), and airway hyper-responsiveness to methacholine (defined as a 20% reduction in FEV_1_ with methacholine concentration ≤12·5 mg/mL). All regressions used quantitative variables as linear predictors, and analyses were performed using R 3.5.1.

### Role of the funding source

GlaxoSmithKline was involved in the design and collection of the original genotype and phenotype data for the ECLIPSE and GenKOLS studies. No other funder had any role in study design, data collection, data analysis, data interpretation, or writing of the report. The corresponding author had full access to all data in the study and had final responsibility to submit for publication.

## Results

**Figure 1:**
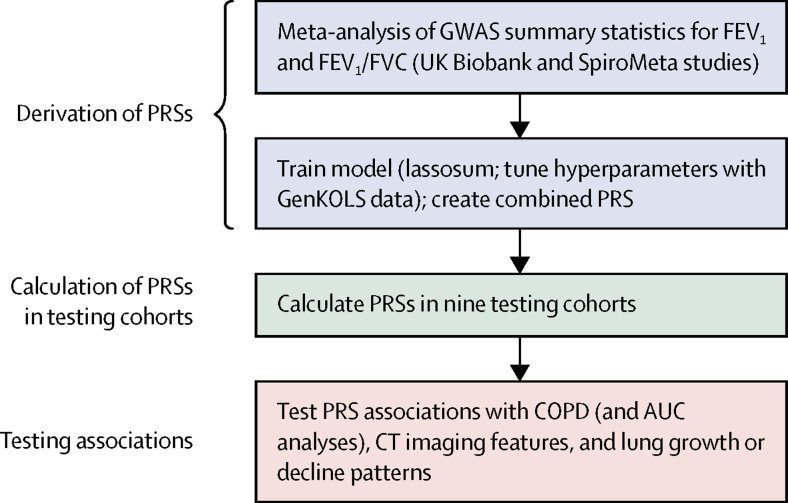
Study design AUC=area under the curve. COPD=chronic obstructive pulmonary disease. FVC=forced vital capacity. GWAS=genome-wide association study. PRS=polygenic risk score.

A schematic of the study design is shown in figure 1. We used GWAS summary statistics of approximately 7·4 million single nucleotide polymorphisms (SNPs) from the UK Biobank (n=321 047) and SpiroMeta (n=79 055) as weights for the development of polygenic risk scores (appendix pp 20–21).^7^ After filtering on variants present in test cohorts and applying a penalised regression framework, our final individual polygenic risk score for FEV_1_ contained 1·7 million SNPs and the individual polygenic risk score for FEV_1_/FVC contained 1·2 million SNPs with non-zero effect sizes; 455 432 SNPs were present in both scores (appendix pp 19–20). The selected shrinkage was 0·9, with a selected λ of 0·0013 for the FEV_1_ polygenic risk score and 0·0016 for the FEV_1_/FVC polygenic risk score. Using logistic regression, we generated a combined model: PRS_Combined_=0·43847 × PRS_FEV1_ + 0·58833 × PRS_FEV1/FVC_, in which PRS is polygenic risk score. In GenKOLS, individual polygenic risk scores for FEV_1_ and FEV_1_/FVC explained 32% and 31% of their corresponding phenotypic variance, respectively. Individual and combined polygenic risk scores trained in COPDGene non-Hispanic white participants and tested in GenKOLS performed similarly (appendix p 22). The combined FEV_1_ and FEV_1_/FVC polygenic risk score included approximately 2·5 million SNPs and was not correlated to smoking pack-years (appendix p 23). Characteristics of additional studies, which include COPD case-control studies, population cohorts, and multiple ethnic groups, are shown in table 1.

**Table 1:** Characteristics of testing cohorts

	**n**		**Age, years**		**Pack-years of smoking**		**FEV**_1_**% predicted**		**FEV**_1_**/FVC ratio**		**Female**		**Ever smokers**	
	Controls	Cases	Controls	Cases	Controls	Cases	Controls	Cases	Controls	Cases	Controls	Cases	Controls	Cases
COPDGene AA participants	1556	910	52·84 (6·01)	58·60 (8·15)	36·11 (19·10)	42·69 (23·48)	98·85 (12·54)	55·50 (19·34)	0·80 (0·06)	0·55 (0·14)	637 (40·9%)	408 (44·8%)	1556 (100·0%)	910 (100·0%)
COPDGene NHW participants	2110	3065	59·18 (8·64)	64·38 (8·28)	37·34 (20·14)	54·88 (27·13)	98·09 (10·98)	52·62 (19·61)	0·78 (0·05)	0·51 (0·14)	1086 (51·5%)	1384 (45·2%)	2110 (100·0%)	3065 (100·0%)
CHS AA participants	258	116	72·68 (5·13)	72·12 (4·85)	10·42 (17·99)	24·32 (29·26)	106·13 (16·61)	61·50 (14·55)	0·77 (0·05)	0·58 (0·10)	194 (75·2%)	51 (44·0%)	130 (50·4%)	95 (81·9%)
CHS EA participants	1480	609	71·78 (5·10)	72·31 (5·22)	10·20 (18·97)	32·48 (30·81)	101·42 (13·04)	60·55 (15·89)	0·76 (0·044)	0·57 (0·11)	1002 (67·7%)	331 (54·4%)	627 (42·4%)	458 (75·2%)
ECLIPSE	147	1713	57·32 (9·55)	63·64 (7·10)	31·01 (25·94)	50·50 (27·47)	106·49 (20·86)	47·34 (15·75)	0·78 (0·15)	0·44 (0·12)	63 (42·9%)	563 (32·9%)	147 (100·0%)	1713 (100·0%)
GenKOLS	692	836	55·43 (9·74)	65·44 (10·10)	19·40 (13·61)	31·88 (18·62)	95·99 (9·11)	50·71 (17·59)	0·80 (0·04)	0·51 (0·13)	338 (48·8%)	328 (39·2%)	692 (100·0%)	836 (100·0%)
Kangwon University	1600	794	47·11 (10·47)	67·69 (9·24)	7·77 (12·03)	36·93 (25·18)	101·20 (11·42)	54·94 (16·02)	0·82 (0·058)	0·50 (0·12)	546 (34·1%)	63 (7·9%)	769 (48·1%)	736 (92·7%)
Lung Health Study	946	1809	47·62 (6·82)	48·99 (6·62)	38·09 (18·05)	42·05 (18·35)	84·74 (2·84)	70·59 (6·58)	0·66 (0·04)	0·62 (0·06)	332 (35·1%)	667 (36·9%)	946 (100·0%)	1809 (100·0%)
MESA AA participants	645	115	64·75 (9·16)	68·42 (9·05)	8·38 (14·96)	20·62 (21·11)	102·07 (14·29)	63·62 (13·05)	0·79 (0·05)	0·58 (0·10)	370 (57·4%)	36 (31·3%)	356 (55·2%)	89 (77·4%)
MESA Chinese participants	422	31	64·28 (9·41)	69·03 (9·01)	3·73 (10·58)	8·02 (15·70)	104·55 (13·87)	65·14 (13·59)	0·78 (0·05)	0·60 (0·09)	213 (50·5%)	14 (45·2%)	115 (27·3%)	10 (32·3%)
MESA Hispanic participants	613	62	63·65 (9·69)	68·63 (9·36)	5·27 (12·36)	16·62 (25·24)	100·54 (12·86)	63·01 (15·16)	0·79 (0·04)	0·59 (0·11)	335 (54·6%)	21 (33·9%)	293 (47·8%)	47 (75·8%)
MESA NHW participants	948	208	65·26 (9·63)	69·27 (8·95)	10·32 (19·93)	30·53 (36·32)	99·22 (11·99)	65·28 (12·70)	0·77 (0·05)	0·60 (0·09)	508 (53·6%)	97 (46·6%)	522 (55·1%)	168 (80·8%)
NETT/NAS	429	371	69·86 (7·50)	67·45 (5·77)	40·69 (27·79)	66·25 (30·66)	100·02 (13·26)	28·13 (7·40)	0·79 (0·05)	0·32 (0·06)	0	135 (36·4%)	429 (100·0%)	371 (100·0%)
RS cohort 1	911	127	79·05 (4·54)	80·34 (4·99)	13·22 (18·81)	26·90 (24·05)	102·77 (17·37)	63·44 (11·36)	0·78 (0·05)	0·61 (0·07)	538 (59·1%)	55 (43·3%)	582 (63·9%)	104 (81·9%)
RS cohort 2	867	96	72·10 (4·88)	73·73 (5·55)	12·93 (19·57)	34·11 (25·91)	101·72 (15·37)	62·27 (12·25)	0·79 (0·05)	0·6 (0·08)	472 (54·4%)	41 (42·7%)	545 (62·9%)	88 (91·7%)
RS cohort 3	1737	131	62·03 (5·38)	63·43 (6·14)	11·56 (17·09)	35·31 (27·14)	101·70 (14·73)	64·55 (12·74)	0·79 (0·05)	0·6 (0·08)	997 (57·4%)	61 (46·6%)	1107 (63·7%)	116 (88·5%)
SPIROMICS NHW	537	988	62·95 (9·00)	65·74 (7·62)	44·76 (26·36)	56·11 (28·78)	90·90 (13·45)	45·90 (16·74)	0·75 (0·05)	0·49 (0·13)	287 (53·4%)	432 (43·7%)	537 (100·0%)	988 (100·0%)

Data are n, mean (SD), or n (%). In total, polygenic risk scores were tested in 27 879 participants (15 898 controls and 11 981 cases). AA=African American. NHW=non-Hispanic white. EA=European ancestry. CHS=Cardiovascular Health Study. NAS=Normative Aging Study. RS=Rotterdam Study.

**Figure 2:**
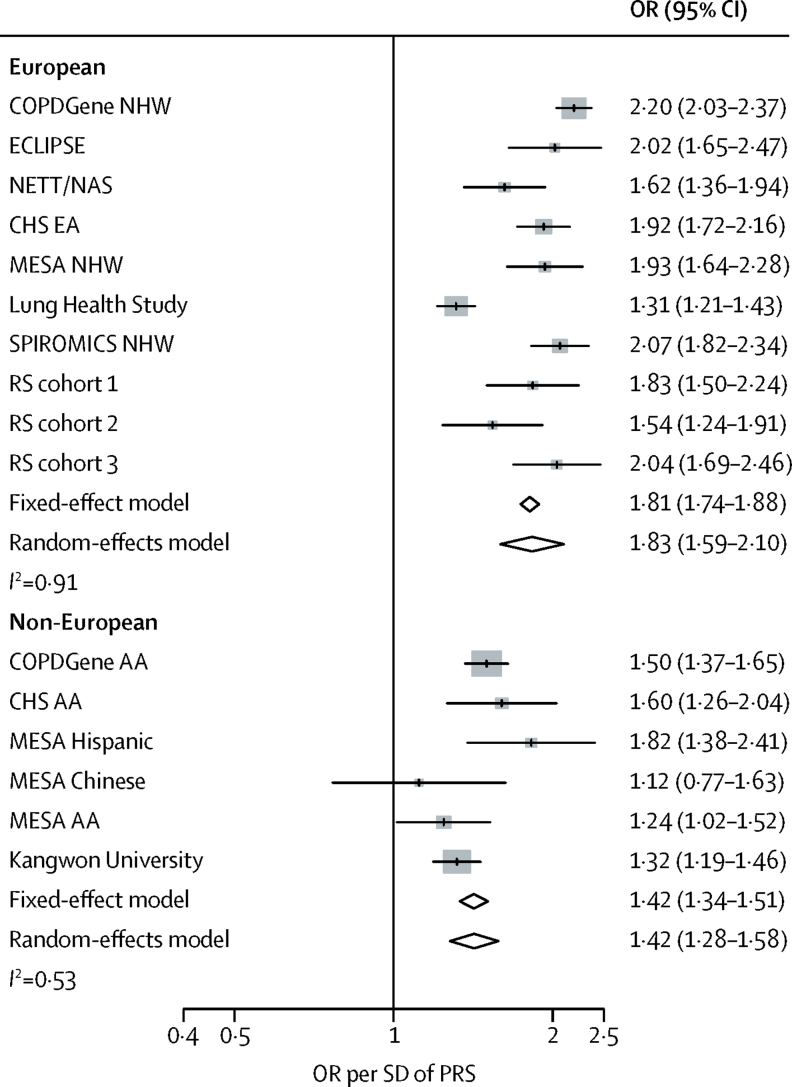
Association of combined PRS with chronic obstructive pulmonary disease AA=African American participants. CHS=Cardiovascular Health Study. EA=European ancestry. NAS=Normative Aging Study. NHW=non-Hispanic white participants. OR=odds ratio. PRS=polygenic risk score. RS=Rotterdam Study.

The results for the primary outcome measure for individual cohorts are shown in figure 2. The combined polygenic risk score was associated with COPD in Europeans (odds ratio [OR] per SD of the score 1·81 [95% CI 1·74–1·88], p=1·8 × 10^−187^). In non-Europeans, effects were generally weaker, although still significant for the majority of studies (1·42 [1·34–1·51], p=2·3 × 10^−29^). We found evidence of study heterogeneity (Europeans *I*^2^=0·91, non-Europeans *I*^2^=0·53); however, random-effects and fixed-effects models generally yielded similar results (figure 2). A funnel plot to assess for systematic bias demonstrated symmetrically distributed effects across studies (appendix p 24). To examine the effect of weights, we evaluated the performance of a combined polygenic risk score using a simple sum (unweighted), which performed similarly to the weighted model (data not shown).

The separate FEV_1_ and FEV_1_/FVC polygenic risk scores were both associated with GOLD spirometry grade in the COPDGene study (appendix pp 25–26). In this study, participants with PRISm had a higher average FEV_1_ polygenic risk score than participants with GOLD 0 or 1 grades (non-Hispanic white participants, PRISm *vs* GOLD 0 p=4·1 × 10^−12^, PRISm *vs* GOLD 1 p=8·7 × 10^−5^ African American participants, PRISm *vs* GOLD 0 p=2·8 × 10^−5^, PRISm *vs* GOLD 1 p=0·01).

**Figure 3:**
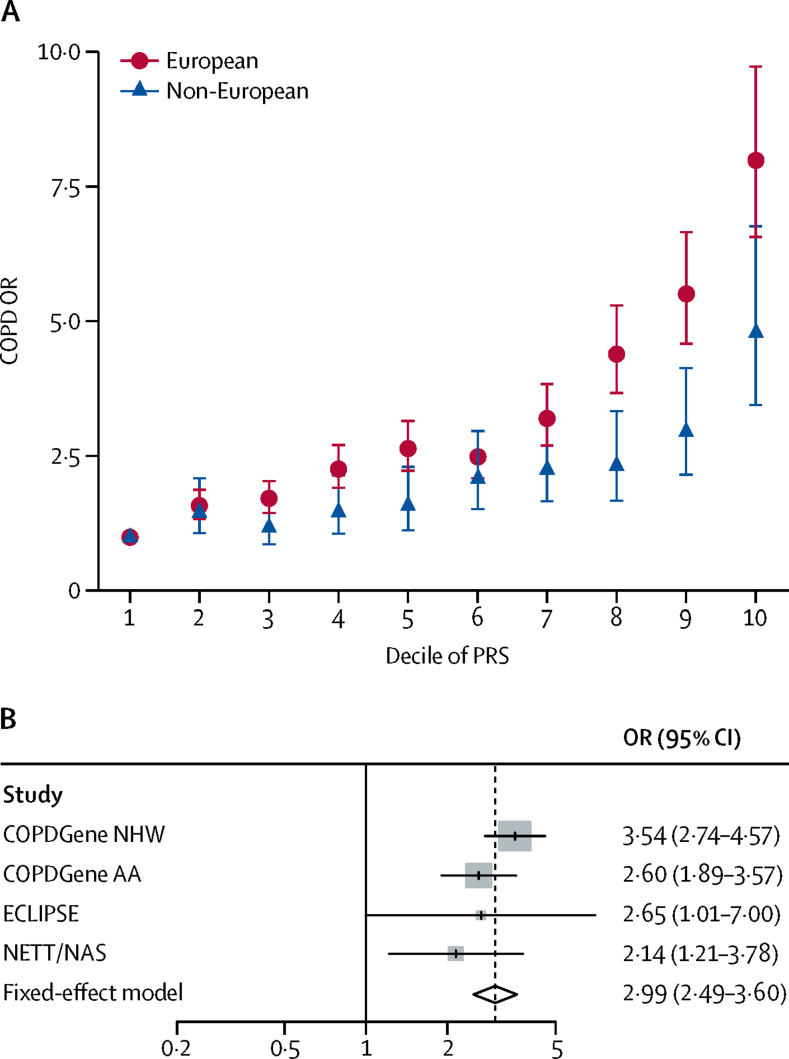
Analysis of OR for COPD by PRS decile (A) ORs for COPD for those in each decile of the PRS in comparison with the first decile in European cohorts and non-European cohorts. Data are shown as ORs with 95% CIs. (B) A secondary meta-analysis comparing COPD risk for participants in the tenth decile with those in the middle tertile of the combined PRS. COPD=chronic obstructive pulmonary disease. AA=African American. NAS=Normative Aging Study. NHW=non-Hispanic white participants. OR=odds ratio. PRS=polygenic risk score.

To further illustrate COPD risk for individuals with different polygenic risk score values, we grouped participants in each study by combined polygenic risk score deciles.^17^ Comparing European-ancestry individuals with the highest scores (tenth decile) to those with the lowest scores (first decile), the OR for COPD was 7·99 (95% CI 6·56–9·72; figure 3A). Comparing non-European-ancestry individuals with the highest scores (tenth decile) to those with the lowest scores (first decile), the OR for COPD was 4·83 (3·45–6·77). Comparing participants in the tenth decile of risk to the middle tertile of risk in the COPDGene, ECLIPSE, and NETT/NAS studies, the OR across these three cohorts was 2·99 (2·49–3·60; figure 3B). If we had theoretically screened individuals in the tenth decile of the polygenic risk score, 18% of individuals with COPD from the CHS European ancestry general population and 14% from the COPDGene non-Hispanic white cohorts would have been detected.

For COPDGene non-Hispanic white participants, we found an AR% of 54% and a PAR% of 57%. We estimated that targeting smoking cessation efforts (assuming complete efficacy) at the 14% of individuals with COPD in the top risk score decile would result in an overall reduction in COPD incidence of 7%. By contrast, targeting the same number of individuals in the lowest decile, among whom the prevalence of COPD is lower, would result in an overall reduction of COPD of 3%. For African American participants, the corresponding PAR% was 22%, and the reduction in total COPD cases for the highest and lowest deciles was 3% and 1%, respectively. A plot of the distribution of polygenic risk and pack-years of smoking for COPD cases and controls among COPDGene non-Hispanic white participants is provided in the appendix (p 27).

The combined polygenic risk score was positively associated with both severe and frequent exacerbations^23^, ^24^ after adjusting for age, sex, and pack-years. However, this association did not persist after adjusting for FEV_1_ and FEV_1_/FVC (appendix p 12). R code and model weights are available online.

**Figure 4:**
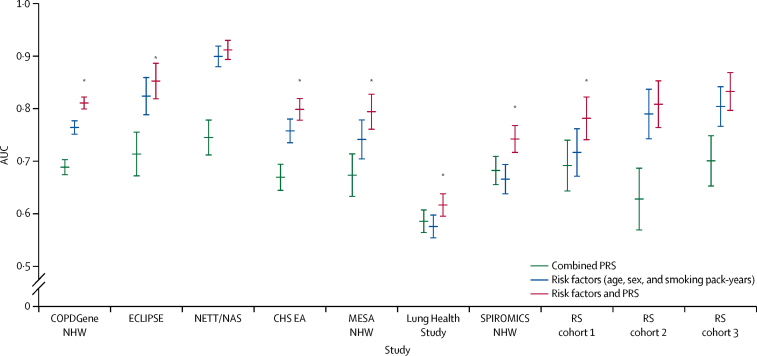
AUC for predicting chronic obstructive pulmonary disease of models including PRS alone, clinical risk factors alone, or both PRS and clinical risk factors AUCs with 95% CIs are shown. Only European cohorts are included in this figure. Asterisks indicate the models including PRS and clinical risk factors for which the AUCs were significantly different from those with clinical risk factors alone (Bonferroni-corrected significance level of 0·005; appendix pp 14–15). Note that an AUC of 0·5 represents the effect assumed under the null model. AUC=area under the curve. EA=European ancestry. NAS=Normative Aging Study. NHW=non-Hispanic white participants. PRS=polygenic risk score.

We assessed performance of the combined polygenic risk score to predict COPD, and found an AUC of 0·67 (95% CI 0·66–0·68). The predictive ability of a model including the polygenic risk score alone was lower than that of a model including clinical COPD risk factors (age, sex, and smoking pack-years) alone; however, a model incorporating both the polygenic risk score and COPD risk factors performed better than a model containing clinical risk factors alone (AUC 0·80 [0·79–0·81] for polygenic risk score plus clinical factors *vs* 0·76 [0·75–0·76] for clinical factors alone; p=1·3 × 10^−41^; figure 4, appendix pp 14–15). Similar results were obtained whether we meta-analysed AUCs using inverse variance weighting or effective sample size weighting (appendix p 13). The predictive performance of each polygenic risk score, including the separate FEV_1_ and FEV_1_/FVC polygenic risk scores, in all testing cohorts is shown in the appendix (p 28).

Because the polygenic score was externally derived (from the UK Biobank and SpiroMeta) and not derived from the test cohorts, we sought to determine the performance of an externally derived clinical score in the COPDGene and ECLIPSE cohorts. We observed that the externally derived clinical risk score performed worse than the polygenic risk score, and that combining both scores resulted in a superior performance to either score alone (appendix p 29). Performance measures (positive predictive value, negative predictive value, sensitivity, and specificity) of the clinical risk score and polygenic risk score are shown in the appendix (p 16). Sensitivities were similar for the clinical risk score (0·85) and polygenic risk score (0·82), whereas the specificity was higher for the polygenic risk score (0·40) than the clinical risk score (0·21). The sensitivity of both the CRS and PRS together was 0·82, but the specificity was 0·61.

We tested for association between the polygenic risk score and CT imaging phenotypes, including quantitative emphysema (%LAA less than –950 HU, Perc15), airway phenotypes (Pi10 and WAP), gas trapping, qualitative emphysema, and local histogram patterns. We found significant associations between the polygenic risk score and %LAA less than –950 HU, Perc15, Pi10, WAP, and gas trapping; the association with qualitative emphysema did not reach significance after correcting for multiple testing (appendix p 24). The association between polygenic risk score and %LAA less than –950 HU was attenuated after adjusting for FEV_1_ % predicted, but the association between polygenic risk score and greater WAP remained significant after this adjustment; the clinical significance of these findings is unclear (appendix p 14). The polygenic risk score was tested for association with local histogram patterns in the COPDGene and ECLIPSE studies; meta-analysis of these two studies showed that the polygenic risk score was positively associated with panlobular and centrilobular local histogram patterns of emphysema, and negatively associated with a normal local histogram pattern (table 2). In COPDGene NHW participants, the polygenic risk score was associated with all ten CT subtypes, and for six subtypes the association met Bonferroni-corrected level of significance (p<0·0025) when compared with normal imaging (CT subtype 1; table 3). The combined polygenic risk score was also tested for association with visual emphysema severity in the COPDGene NHW population, and every SD increase in the combined polygenic risk score was associated with an OR of 1·20 (95% CI 1·13–1·27, p=2·7 × 10^−10^) of being in a higher visual emphysema category (appendix p 18).

**Table 2:** Association of the combined FEV_1_ and FEV_1_/forced vital capacity polygenic risk score with local histogram patterns of emphysema

	**β (CI)**	**R^2^**	**p value**
Normal	−0·012 (−0·022 to −0·0042)	0·32	2·0 × 10^−11^
Panlobular	0·0045 (0·0017 to 0·0084)	0·064	1·1 × 10^−12^
Mild centrilobular	0·0027 (−0·0015 to 0·0074)	0·16	0·0012
Moderate centrilobular	0·0084 (0·002 to 0·016)	0·27	2·0 × 10^−10^
Severe centrilobular	0·0029 (−0·00031 to 0·0068)	0·078	4·1 × 10^−5^

n=7600 in all categories. CIs are Bonferroni-corrected. β coefficients are from Tobit regression and indicate the quantitative increase in a given CT imaging measure for one SD increase in the polygenic risk score on the latent uncensored variable. Participants from COPDGene and ECLIPSE were included, and reported values reflect fixed-effects meta-analysis results. The same individuals can have multiple local histogram patterns of emphysema, so the number of participants in each category are not mutually exclusive.

**Table 3:** Association of the combined FEV_1_ and FEV_1_/forced vital capacity polygenic risk score with CT imaging subtypes in the COPDGene study^49^

	**Description of phenotype**	**COPDGene non-Hispanic white participants**		
		n	Odds ratio (CI)	p value
Subtype 1	Normal imaging	1597	1 (ref)	..
Subtype 2	Paraseptal emphysema	972	1·3 (1·1–1·4)	5·43 × 10^−6^
Subtype 3	Bronchial airway disease	437	1·4 (1·1–1·6)	4·73 × 10^−7^
Subtype 4	Small airway disease	255	1·4 (1·1–1·7)	1·47 × 10^−5^
Subtype 5	Mild centrilobular emphysema	1100	1·2 (1·1–1·4)	1·13 × 10^−5^
Subtype 6	Moderate-to-severe centrilobular emphysema: upper lobe dominant	186	1·3 (0·99–1·7)	0·00742
Subtype 7	Moderate-to-severe centrilobular emphysema: lower lobe dominant	23	1·8 (0·8–3·7)	0·0322
Subtype 8	Moderate-to-severe centrilobular emphysema: diffuse	346	1·5 (1·2–1·9)	2·01 × 10^−7^
Subtype 9	Discordant: visual emphysema without quantitative emphysema	311	1·4 (1·1–1·7)	1·02 × 10^−5^
Subtype 10	Discordant: quantitative emphysema without visual emphysema	109	1·3 (0·9–1·9)	0·0496

Multiple logistic regressions were performed in which presence of each CT subtype was compared to normal imaging (subtype 1) as a reference group. CIs are Bonferroni-corrected.

McGeachie and colleagues^18^ reported four patterns for lung function growth and decline in the CAMP study. Children with persistent asthma and reduced growth of lung function had increased risk for COPD in early adulthood, based on GOLD post-bronchodilator spirometry criteria. We tested for association between the polygenic risk score and patterns of lung function growth and decline. After adjusting for age, sex, height, baseline FEV_1_, % bronchodilator response, and airway hyper-responsiveness, one SD increase in the polygenic risk score was associated with having a pattern of reduced lung growth (OR 1·07 [95% CI 1·02–1·11], p=0·002; appendix p 33). The polygenic risk score was also positively associated with COPD affection status as defined by FEV_1_/FVC <0·7 at the end of the CAMP study (p=0·0005).

## Discussion

In this analysis, we developed a polygenic risk score using GWAS summary statistics from more than 400 000 participants, and used it to predict the diagnosis of COPD in nine population-based and case-control cohorts. This score used more variants and larger sample sizes than previous studies, and was tested in a greater number of validation cohorts.^7^, ^11^, ^14^, ^16^ The score was also associated with CT imaging phenotypes and patterns of reduced lung growth that could predispose individuals to COPD.

The predictive performance of our combined FEV_1_ and FEV_1_/FVC polygenic risk score for COPD compares favourably with previous studies. A GWAS of approximately 12 000 individuals was used to develop an unweighted 30-variant genetic risk score that had moderate (AUC 0·58) predictive power for COPD.^14^ In a GWAS including nearly 49 000 UK Biobank participants, a 95-variant risk score had a 3·7-times greater risk of COPD when comparing the highest and lowest deciles of the risk score.^11^ In a larger GWAS^7^ including more than 400 000 individuals that tested a 279-variant weighted genetic risk score of lung function in multiple external validation cohorts, the highest decile was associated with 4·73-times greater odds of COPD compared with the lowest decile. These data suggest that a larger GWAS with more accurate weights, and including more variants, improves the predictive performance of a genetic risk score. Consistent with this suggestion, our combined polygenic risk score was associated with a 7·99-times increase in the odds of COPD when comparing the highest and lowest deciles of the risk score in European populations. Furthermore, the meta-analysed AUC for our polygenic risk score was higher (COPDGene, ECLIPSE, NETT/NAS polygenic risk score AUC 0·68 [95% CI 0·65–0·70]) than the AUC for the 279-variant genetic risk score reported by Shrine and colleagues (AUC 0·58 [0·56–0·61], p=2·7 × 10^−41^).^7^

This improved performance could reflect several factors: the number of variants included (around 1·2–1·7 million), inclusion of variants not reaching genome-wide significance, large GWAS sample size (>400 000 individuals), and GWAS-derived variant weights. By applying a regularised regression method to include variants not reaching genome-wide significance and combining two lung function parameters, our study used the same GWAS as Shrine and colleagues and achieved a substantial improvement in prediction. To our knowledge, our study is the first to apply genome-wide polygenic scores to respiratory disease. The application of this method to other populations remains to be evaluated.

Our polygenic risk score has the potential to identify individuals at a markedly increased risk of COPD. In Europeans, for every SD increase in the combined FEV_1_ and FEV_1_/FVC polygenic risk score, we observed an OR for COPD of 1·81; by comparison, for every 10 pack-years of smoking, an OR of between 1·16 and 1·28 has been reported.^53^ On the basis of these previous estimates of the effects of smoking on COPD risk, being in the tenth decile of polygenic risk is similar to having 84–140 pack-years of smoking history. Furthermore, the polygenic risk score was not correlated with smoking pack-years, which suggests that it provides information regarding COPD risk that is independent of smoking history. We estimated that a reduction in smoking to 20 pack-years or less in COPDGene non-Hispanic white participants in the highest polygenic risk categories would result in a 7% reduction in COPD, versus 3% in the lowest risk category. These results are based on observational data, and in addition are highly dependent on estimates of population attributable risk. Studies in larger, population-based cohorts, including those with less cigarette smoke exposure, will be needed to confirm these results.

The only routine genetic screening recommended in COPD is for α1 antitrypsin deficiency, which is present in about 1% of individuals with COPD.^54^, ^55^ Our score identifies 10% of the population at around three-times greater odds for COPD compared with the middle tertile of the population, and around 15–20% of individuals who will develop COPD. Thus, at a young age, we could potentially identify individuals at risk for COPD and implement strategies to optimise lung health. Although the major preventive measure, avoidance of cigarette smoking, is recommended for all individuals, a study in individuals with α1 antitrypsin deficiency suggests that knowledge of genetic susceptibility to COPD can motivate smoking cessation attempts.^56^

In theory, obtaining a polygenic risk score in clinical practice involves obtaining a DNA sample and testing a set of genome-wide genetic markers, which would only need to be done once in a person's lifetime and at a cost of less than US$100. Millions of individuals already possess these data through direct-to-consumer testing, and genome-wide genotype data might become part of the future medical record. We observed that clinical factors alone had a higher AUC than the polygenic risk score, and that adding clinical factors to the polygenic risk score significantly improved the AUC (and vice versa). One factor in the superior performance of the clinical factors is that, by contrast with the polygenic risk score (which was trained on an external dataset), the predictive value of the clinical factors was measured from the same cohort, potentially overestimating the performance of the clinical risk factors. For example, we observed that a clinical risk score derived from age, sex, and smoking pack-years in the UK Biobank cohort actually performed worse than the polygenic risk score in two of our cohorts. In addition, the availability of the polygenic risk score throughout the life course suggests that polygenic risk score might be more helpful earlier in life than a clinical risk score, or in scenarios in which up-to-date clinical information is not available (eg, smoking history), such as in some population studies. Although further studies of specific interventions are needed before clinical application, our data suggest that the greatest immediate clinical utility of the polygenic risk score could be in younger populations without substantial smoking history, which could lead to implementation of focused prevention strategies. Unlike other omics, such as gene expression, proteomics, or metabolomics, polygenic risk scores can be measured at birth and do not change over an individual's lifetime. Therefore, the polygenic risk score provides an assessment of risk before the occurrence of environmental exposures. As clinical risk factors change throughout an individual's lifetime (ie, age and smoking history), a person's COPD risk could be updated, and targeted intervention strategies could be employed. Further investigation into the clinical utility of polygenic risk scores is needed.

To our knowledge, this is the first report of a statistically significant association between a genetic risk score and COPD exacerbations. This association suggests a shared mechanism between lung function and COPD exacerbations, consistent both with published reports that low lung function is a risk factor for exacerbations,^57^, ^58^ and with this association no longer being observed after adjustment for lung function. The latter observation indicates that the polygenic risk score is unlikely to add utility to the prediction of exacerbations when baseline lung function is already available and incorporated in the prediction model.

Our polygenic risk score was associated with several CT imaging phenotypes, including greater quantitative emphysema (%LAA less than –950 HU and Perc15), measures of airway wall thickness (Pi10 and WAP), gas trapping, and local histogram patterns of emphysema. Although genetic variants have been previously reported to associate with many of these CT imaging measures,^8^, ^59^, ^60^, ^61^ previous reported variants had varying effect sizes and directions. In 2019, a report of a genetic risk score for lung function identified associations with CT phenotypes.^16^ Our polygenic risk score builds on these results by using a genome-wide polygenic score and testing of additional phenotypes in multiple cohorts. Our findings were largely consistent across studies, with the exception of NETT/NAS, which had a narrower range of phenotypes and did not use a standard CT protocol. Compared with previous reports of genetic risk score associations with CT phenotypes, our polygenic risk score had larger effect sizes; for example, the association with WAP was 0·68 (adjusted CI 0·59–0·77) versus 0·22 (95% CI 0·13–0·32) reported previously.^16^ The associations with WAP and Pi10 are notable because previous genetic association studies have not shown genome-wide significant association of single variants with measures of airway wall thickness.^8^ The association between polygenic risk score and %LAA less than –950 HU was attenuated after adjusting for FEV_1_ % predicted, but the association between polygenic risk score and WAP remained significant after this adjustment; the significance of these findings is unclear, and requires additional, systematic investigation across multiple cohorts. We found an association between the polygenic risk score and a broad range of CT phenotypes, suggesting that the combined polygenic risk score could capture much of the heterogeneity measured by CT imaging. Local histogram emphysema phenotypes are associated with poor lung function, dyspnoea, and quality of life^48^; we observed an association of the polygenic risk score with panlobular and centrilobular emphysema phenotypes. Visual emphysema severity scores based on Fleishner guidelines predict mortality in COPD,^50^ and we observed that a higher polygenic risk score was associated with higher levels of visual emphysema. Visual and quantitative emphysema have been shown to have both overlapping and independent associations with genetic variants.^62^ The association between the polygenic risk score and qualitative and quantitative measures of emphysema suggests that the polygenic risk score is predictive of a wide range of early and late lung structural changes. This finding is important because lung structural changes detected by CT might precede and be discordant with spirometric changes;^63^ in this context, the polygenic risk score could have a role in reducing the radiation and economic burden of large-scale CT phenotyping for early diagnosis. Thus, the combined polygenic risk score might account for the wide range of heterogeneity in individuals at risk for or with varying phenotypes of COPD.

The polygenic risk score was associated with patterns of reduced lung growth in children with asthma, and with incident COPD among participants aged 23–30 years at the conclusion of 16–18 years of observation. Impaired or reduced lung growth during development may predispose individuals to COPD. These data are consistent with genetic association studies of COPD that find associated variants enriched (ie, statistically more likely to occur) in regions of the genome that are important for gene regulation in the fetal lung.^10^, ^11^ These findings are also consistent with the study by Lange and colleagues,^64^ which found that a substantial proportion of individuals with COPD have low lung function in early adulthood. When patterns of normal or reduced lung growth were used to stratify participants in the CAMP study, 18% of individuals with reduced patterns of lung growth developed COPD compared with 3% of individuals with a normal pattern of lung growth.^18^ Thus, the polygenic risk score is capturing combinations of genetic variants responsible for impaired lung growth and susceptibility to COPD.

Genetic determinants of COPD susceptibility and heterogeneity could be shared or distinct. Our study shows evidence of shared susceptibility in lung growth and imaging patterns, and potentially in exacerbations; major determinants of symptoms, decline, and exacerbations might be different from determinants of susceptibility. Genetic studies of these specific phenotypes, as well as elucidation of the specific functional components underlying genetic risk, could help address some of these questions. Recent work into partitioning of genetic risk scores in diabetes^65^ suggests that future polygenic risk scores could be used to identify genetic sources of heterogeneity.

Our study is based on cross-sectional lung function measures in cohorts of different ages and cigarette smoking exposure. Estimates of the prevalence and absolute risk of COPD will depend on the specific characteristics of the cohort. Our study does not address the challenging issue of longitudinal measures. For example, lung function decline is a heritable trait,^66^ yet no studies have identified and replicated genome-wide significant variants associated with lung function decline. Furthermore, the greatest decline might be seen in those with the highest lung function.^67^ Individual genetic variants associated with cross-sectional lung function measures have generally not been predictive of lung function decline.^68^ Our risk score associates with reduced lung growth, and also with emphysema patterns characteristic of older smoking adults with severe COPD. Whether the latter includes structural abnormalities present in younger age, or is due to dysregulated pathways in adults, is still unclear. It would be interesting to observe whether individuals with reduced patterns of lung growth have structural abnormalities detectable on CT imaging, such as emphysema or thickened airways. However, a limitation of the CAMP study is that imaging data were not obtained. Longitudinal studies of lung function and imaging phenotypes and elucidation of the specific functional components underlying genetic risk could help to address some of these questions.

We focused on the analytic and clinical validity of the polygenic risk score in predicting COPD; evaluation of the impact of screening for COPD using such a risk score was beyond the scope of this study. Despite the magnitude of effect sizes observed in this study, large ORs do not always translate into effective screening tests.^69^ The American College of Physicians and US Preventive Task Force guidelines recommend against screening with spirometry until symptoms develop,^70^, ^71^ yet a substantial number of individuals with COPD are undiagnosed and under-report symptoms,^72^, ^73^ and these individuals could lose substantial lung function before the time of diagnosis.^74^, ^75^ Therefore, future studies could evaluate whether the use of the polygenic risk score could help identify patients at greater risk of COPD and reduce underdiagnosis. Assessing the potential benefits and harms of implementing polygenic risk scores for COPD screening is challenging,^69^, ^76^ and warrants formal investigation.

Although we demonstrate some shared genetic architecture between susceptibility, imaging phenotypes, and exacerbations, the individual genetic determinants of these traits might differ. Genetic studies of these specific phenotypes, and elucidation of the specific functional components underlying genetic risk, could help address some of these questions. One major limitation to the clinical application of our polygenic risk score to COPD treatment is the lack of effective interventions to preserve lung health. Apart from smoking cessation, other potential strategies, such as avoidance of air pollution or other environmental risk factors, adequate nutrition (eg, vitamin D supplementation^77^), and bronchodilators^78^ are not well supported by evidence. However, it is possible that polygenic risk scores could be used for trial selection to reduce heterogeneity. Furthermore, the role of polygenic risk scores for understanding COPD pathogenesis is an important area in need of exploration.

COPD is a worldwide disease, but most individuals studied have been of European ancestry. Although the highest decile of the combined polygenic risk score was associated with a more than four-times higher odds of COPD compared with the lowest decile in non-Europeans, the current study was not designed to address the important disparities in the quality and availability of genetic data in European compared with non-European populations. The development of polygenic risk scores in multi-ethnic populations using appropriate methods will be crucial for the widespread implementation of precision medicine, and to prevent widening of health-care disparities.^79^ We noted heterogeneity, which in some cases was likely to be due to characteristics of the specific cohort. For example, the LHS study enrolled only smokers with mild airflow obstruction, leading to the lowest lung function in controls and the highest lung function in cases of the studied population. Our study did not include rare variants or non-additive genetic models. Previous studies suggest that α1 antitrypsin (*SERPINA1*) variants^44^, ^55^ and other rare variants^80^ might be important. Our study also did not explore the role of other omics or molecular biomarkers (eg, fibrinogen, interleukins). Because these data can vary over time, careful study of these factors in longitudinal datasets is likely to be needed.

The past decade has seen important progress in genomic medicine. Leveraging recent large GWASs, we developed a polygenic risk score that has substantial predictive power and complements clinical risk factors for COPD across nine different cohorts. The polygenic risk score is related to a range of imaging phenotypes, including emphysema patterns, as well as reduced lung growth. These findings could have important implications for understanding the mechanisms underlying COPD and provide future opportunities for prevention and early intervention, as genomics become more widely adopted in health care.
