# Correction to *Lancet Respir Med* 2020; 8: 696–708

**DOI:** 10.1016/S2213-2600(24)00326-6

**Published:** 2024-11

*Moll M, Sakornsakolpat P, Shrine N, et al. Chronic obstructive pulmonary disease and related phenotypes: polygenic risk scores in population-based and case-control cohorts.* Lancet Respir Med *2020; **8:** 696–708*—In this Article, a subset of participants from the SPIROMICS cohort were included in the study who were later found to have not provided consent for genetics or to have withdrawn consent for genetics. SPIROMICS I enrolled participants from 2010 to 2016 and performed up to three follow-up visits up to 2016. During SPIROMICS I, the consent form included several questions, including one on genetics. At each follow-up visit, the consent form was repeated and blood was drawn. This process yielded a complex set of consent flags and biospecimens across up to four visits. Unfortunately, the consent flags extracted from consent form questions were not properly reviewed before the isolation of DNA from the stored blood. Therefore, in 2016, some participants who did not consent for genetics, or who had withdrawn consent for genetics, had DNA isolated from blood and were included in the genome-wide association study. To correct the use of SPIROMICS data in this Article, the individuals who did not provide or who withdrew consent (11 controls and 15 cases) were removed and all analyses involving SPIROMICS data were repeated. In figure 2, the odds ratio (OR) for the association of combined polygenic risk score with chronic obstructive pulmonary disease (COPD) was corrected to 2·15 (95% CI 1·88–2·46) for the SPIROMICS non-Hispanic white (NHW) cohort, to 1·82 (1·74–1·89) for the overall fixed-effect model for European cohorts, and to 1·84 (1·60–2·11) for the overall random-effects model for European cohorts. In figure 3A, the ORs for COPD in European cohorts were corrected. In figure 4, the areas under the curve for predicting COPD in the SPIROMICS NHW cohort were corrected. The appendix has also been corrected. None of the findings changed significantly, and the conclusions of the study are unaffected. These corrections have been made to the online version as of Oct 29, 2024.

