# Supplementary material for: Chronic obstructive pulmonary disease and related phenotypes: polygenic risk scores in population-based and case-control cohorts
Source: Lancet Respir Med. 2020 Jul;8(7):696–708. doi: 10.1016/S2213-2600(20)30101-6 (PMC7429152; doi:10.1016/S2213-2600(20)30101-6)
Supplement: Supplementary appendix [file mmc1.pdf]

THE LANCET
Respiratory Medicine

Supplementary appendix

This appendix formed part of the original submission and has been peer reviewed. We post it as supplied by the authors.

Supplement to: Moll M, Sakornsakolpat P, Shrine N, et al. Chronic obstructive pulmonary disease and related phenotypes: polygenic risk scores in population-based and case-control cohorts. *Lancet Respir Med* 2020; **8:** 697–708.

Supplementary Note

Table of Contents

Individual Study Information…………………………………………………………………2
Funding and Acknowledgements……………………………………………………………..5
Supplementary Methods…………………………………………………………......…11
Supplementary Tables
Table S1…………………………………………………………………..……12
Table S2……………………………………………………………………………...13
Table S3……………………………………………………………………………...14
Table S4……………………………………………………………………………...16
Table S5……………………………………………………………………………...17
Table S6……………………………………………………………………………...18
Supplementary Figures
Figure S1…………………………………………………………………………..19
Figure S2…………………………………………………………………………..20
Figure S3…………………………………………………………………………..21
Figure S4…………………………………………………………………………..22
Figure S5…………………………………………………………………………..23
Figure S6…………………………………………………………………………..24
Figure S7…………………………………………………………………………..25
Figure S8…………………………………………………………………………..27
Figure S9…………………………………………………………………………..28
Figure S10……………………………………………………………………..…..29
Figure S11……………………………………………………..…………..30
Figure S12………………………………………………………..……………..33
References……………………………………………………………………………………34

Individual Study Information

This section describes each study and provides details about measurements and genotyping. All participants gave informed consent and study protocols were approved by local Research Ethics Committees and Institutional Review Boards.

COPDGene

COPDGene is a multicenter observational study which primarily consists of smokers with and without COPD[1]. Subjects in COPDGene have at least 10 pack-years of smoking (except for a smaller group of nonsmoking controls, who were excluded from the analyses in this manuscript). Illumina (San Diego, CA) performed genotyping on the HumanOmniExpress array. Genotyping at the Z and S alleles was performed in all subjects. Subjects with severe alpha-1 antitrypsin deficiency were excluded. Imputation was performed the Michigan Imputation Server to the Haplotype Resource Consortium[2] and 1000 Genomes Phase I v3 Cosmopolitan reference panels, for whites and African Americans, respectively. Variants with an r2 value of ≤ 0.3 were removed.

GenKOLS

The Genetics of Chronic Obstructive Lung Disease (GenKOLS) is a single-center case-control study based in Bergen, Norway[3,4]. Subjects with > 2.5 pack years of smoking history were included; severe alpha-1 antitrypsin deficiency and other lung diseases were excluded. The Regional Committee for Medical Research Ethics (REK Vest), the Norwegian Data Inspectorate and the Norwegian Department of Health approved the case–control study. Written informed consent was obtained from all participants. Genotyping was performed using Illumina HumanHap 550 arrays (Illumina, San Diego, CA). Genotype imputation was the Michigan Imputation Server and Haplotype Resource Consortium[2] reference panel.

NETT/NAS

The National Emphysema Treatment Trial (NETT) was a multicenter randomized clinical trial comparing lung-volume-reduction surgery and medical therapy for severe emphysema[5]. All subjects in NETT were former smokers with severe COPD (FEV_1 ≤45% predicted). The Normative Aging Study (NAS) is a longitudinal study of health and aging[6,7]. We included NAS participants with normal spirometry and at least 10 pack-years of cigarette smoking history as control subjects in NETT/NAS. Genotyping for NETT-NAS was performed using the Illumina Quad 610 array (Illumina, San Diego, CA).[8,9] Imputation was performed using the Michigan Imputation Server and Haplotype Resource Consortium[2] reference panel.

ECLIPSE

The Evaluation of COPD Longitudinally to Identify Predictive Surrogate End-points (ECLIPSE) study was a case-control study of smokers with ≥ 10 pack years of smoking history, aged 40-75 years, and without other respiratory diseases.[10] Genotyping was performed using the Illumina HumanHap 550 V3 (Illumina, San Diego, CA). Subjects and markers with a call rate of < 95% were excluded. Imputation was performed using the Michigan Imputation Server and Haplotype Resource Consortium[2] reference panel.

MESA

The Multi-Ethnic Study of Atherosclerosis (MESA) was a longitudinal study of subclinical cardiovascular disease and risk factors that predict progression to clinically overt cardiovascular disease or progression of the subclinical disease.[11] Further details have been previously reported.[12] Genotyping was performed at Affymetrix (Santa Clara, California, USA) and the Broad Institute of Harvard and MIT (Boston, Massachusetts, USA) using the Affymetrix Genome-Wide Human SNP Array 6.0.

CHS

The Cardiovascular Health Study (CHS) is a population-based cohort study of risk factors for coronary heart disease and stroke in adults ≥65 years conducted across four field centers.[13] The original predominantly European ancestry cohort of 5,201 persons was recruited in 1989-1990 from random samples of the Medicare eligibility lists; subsequently, an additional predominantly African-American cohort of 687 persons was enrolled in 1992-1993 for a total sample of 5,888. Blood samples were drawn from all participants at their baseline examination and DNA was subsequently extracted from available samples. Genotyping was performed at the General Clinical Research Center's Phenotyping/Genotyping Laboratory at Cedars-Sinai among CHS participants who consented to genetic testing and had DNA available using the Illumina 370CNV BeadChip system (for European ancestry participants, in 2007) or the Illumina HumanOmni1-Quad_v1 BeadChip system (for African-American participants, in 2010). European ancestry participants were excluded from the GWAS study sample due to the presence at study baseline of coronary heart disease, congestive heart failure, peripheral vascular disease, valvular heart disease, stroke or transient ischemic attack or lack of available DNA. Imputation to the HRC r1.1 2016 panel was performed on the Michigan imputation server. SNPs were excluded for variance on the allele dosage ≤0.01. Pulmonary measures were taken at the 1989-1990 examination for the first cohort and at the 1993-1994 examination for the second cohort. CHS was approved by institutional review committees at each field center and individuals in the present analysis had available DNA and gave informed consent including consent to use of genetic information for the study of cardiovascular disease.

Rotterdam

The Rotterdam study (RS) is a prospective population-based cohort study conducted in a suburb of Rotterdam, Netherlands.[14] The first cohort (RS-I) enrolled 7,983 participants, aged 55 years and over. The second cohort (RS-II) recruited in 2000. The third cohort (RS-III) consisted of 3,932 participants, aged 45 years and over and was recruited in 2006. A total of 6,291 subjects for RS I, 2,157 for RS II and 3,048 for RS III passed genotyping quality control.

SPIROMICS

The Subpopulations and intermediate outcome measures in COPD study (SPIROMICS) study. Participants of the NHLBI SPIROMICS study were 40-80 years of age at baseline with a smoking history ≥20 pack-years. Recruitment included non-smokers, smokers without COPD, mild-moderate COPD, and severe COPD.[15] Genomewide genotyping was performed using the Illumina OmniExpress HumanExome BeadChip using standard techniques in the first 571 subjects with COPD and 175 smoking controls. Imputation was performed against 1000 Genomes reference panels using Impute-v2.30 using a quality cutoff of 0.9.

KWU

The KWU data consisted of Koreans who visited Kang-Won National University Hospital and Seoul National University Gangnam Center. All subjects were genotyped by using their blood with the Korea Biobank Array version 1.0 (Affymetrix customized SNP chip).[16] SNPs were excluded if any of the following conditions were satisfied; the genotype call rate was less than 95%; the Hardy-Weinberg equilibrium (HWE) test gave $P < 1\times10^{-5}$; MAFs were less than 0.05. Also, subjects were discarded if X chromosome homozygosity were between 0.2 and 0.8; genotype call rates were < 95%; or heterozygosity rates of SNPs were outside the average of heterozygosity rate ± 3 standard deviations. Then untyped SNPs were imputed using the Michigan imputation server (https://imputationserver.sph.umich.edu). Only 'not European' or 'mixed' population of Haplotype Reference Consortium release v1.1 were used as a reference. Pre-phasing and imputation were conducted with Eagle2[17] and Minimac4[18] respectively. Imputed SNPs were removed if there were duplicated SNPs; missing genotype rates were larger than 0.05; P-values for HWE were less than 1×10^{-5}; MAFs were less than 0.05. Also, subjects whose identity-by-descent (IBS) was larger than 0.9 and PC scores were outside the median ± 5×IQR were removed. After quality controls, 794 COPD patients and 1600 normal subjects with 4,922,773 SNPs genotyped were analyzed.

LHS

The Lung Health Study (LHS) was initially a randomized clinical trial across 10 clinical centers in North America, that enrolled 5,887 smokers, 35-60 years of age with COPD. Two-thirds were assigned to smoking intervention and one-third to usual care. Further details have been reported.[19,20] Genotyping was performed using Illumina Human660WQuad v.1_A BeadChip. Imputation was performed with the Michigan Imputation Server [18] using the Haplotype Reference Consortium[2] panel. Variants with r2 < 0.5 and minor allele frequency < 1% were removed.

CAMP

The Childhood Asthma Management Program (CAMP) study was a randomized, placebo-controlled trial of inhaled anti-inflammatory therapies in 1,041 mild-to-moderate childhood asthmatics with three phases of observational follow-up; follow up phases included at least annual pre- and post-bronchodilator spirometry. 684 participants had follow up spirometry after 23 years, and two independent

investigators classified individuals as having one of four lung function growth and decline patterns: normal growth, normal growth with early decline, reduced growth, and reduced growth with early decline.[21] Genome-wide SNP genotyping was performed using Illumina's HumanHAP550 Genotyping BeadChip (Illumina, Inc., San Diego, CA). Imputation was performed using Markov Chain Haplotyping software (MaCH) [22] and variants with minor allele frequency < 1% were removed.

Funding and Acknowledgements

The SpiroMeta Consortium: Nick Shrine, Anna L. Guyatt, Chiara Batini, Jing Hua Zhao, Matthias Wielscher, Unerstanding Society Scientific Group, Stefan Weiss, Katherine A. Kentistou, James P. Cook, Jennie Hui, Stefan Karrasch, Medea Imboden, Sarah E. Harris, Jonathan Marten, Stefan Enroth, Shona M. Kerr, Ida Surakka, Veronique Vitart, Terho Lehtimäki, Ralf Ewert, Christian Gieger, Georg Homuth, Peter K. Joshi, Claudia Langenberg, Lars Lind, Jian'an Luan, Anubha Mahajan, Alison Murray, David J. Porteous, Rajesh Rawal, Blair H. Smith, Paul R. H. J. Timmers, Olli T. Raitakari, Mika Kähönen, Ozren Polasek, Ulf Gyllensten, Igor Rudan, Ian J. Deary, Nicole M. Probst-Hensch, Holger Schulz, Alan L. James, James F. Wilson, Beate Stubbe, Eleftheria Zeggini, Marjo-Riitta Jarvelin, Nick Wareham, Caroline Hayward, Andrew P. Morris, David P. Strachan, Ian P. Hall, Martin D. Tobin and Louise V. Wain.

The International COPD Genetics Consortium: *Executive Committee*: James D. Crapo, William MacNee, David Lynch, H. Marike Boezen, Edwin K. Silverman, Jørgen Vestbo. *Members*: Alvar Agusti, Wayne Anderson, Nawar Bakerly, Per Bakke, Robert Bals, Kathleen C. Barnes, R Graham Barr, Terri H. Beaty, Eugene R. Bleecker, Yohan Bossé, Russell Bowler, Christopher Brightling, Marleen de Bruijne, Peter J. Castaldi, Bartolome Celli, Michael H. Cho, Harvey O. Coxson, Ron Crystal, Pim de Jong, Asger Dirksen, Jennifer Dy, Marilyn Foreman, Judith Garcia-Aymerich, Pierre Gevenois, Soumitra Ghosh, Hester Gietema, Amund Gulsvik, Ian P. Hall, Nadia Hansel, Craig P. Hersh, Brian D. Hobbs, Eric Hoffman, Noor Kalsheker, Hans-Ulrich Kauczor, Woo Jin Kim, Deog Kyeom Kim, Tarja Laitinen, Diether Lambrechts, Sang-Do Lee, Augusto A. Litonjua, David A. Lomas, Stephanie J. London, Daan W. Loth, Sharon M. Lutz, Merry-Lynn McDonald, Deborah A. Meyers, John D. Newell, Borge G. Nordestgaard, George T. O'Connor, Ma'en Obeidat, Yeon-Mok Oh, Peter D. Paré, Massimo Pistolesi, Dirkje S. Postma, Milo Puhan, Elizabeth Regan, Stephen S. Rich, Joon Beom Seo, Andrea Short, David Sparrow, Berend Stoel, David P. Strachan, Nicola Sverzellati, Ruth Tal-Singer, Gerben ter Riet, Yohannes Tesfaigzi, Martin D. Tobin, Edwin J.R. Van Beek, Bram van Ginneken, Claus F. Vogelmeier, Louise V. Wain, Adam Wanner, George Washko, Els Wauters, Emiel FM Wouters, Robert P. Young, and Loems Zeigler-Heitbrock.
The ICGC extends special thanks to Nora Franceschini, Kari North, Steve Rich, Xin-Qun Wang, Andre Uitterlinden, Bruno Stricker, Arfan Ikram, Megan Hardin, Gus Litonjua, Nick Locantore, Josée Dupuis, Elizabeth Ampleford, Eugene Bleecker, Yeon- Mok Oh, Shuguang Leng, Bruce Psaty, Susan Heckbert, and Jerry Rotter.

COPDGene Funding and Acknowledgements

Grant Support and Disclaimer

The project described was supported by Award Number U01 HL089897 and Award Number U01 HL089856 from the National Heart, Lung, and Blood Institute. The content is solely the responsibility of the authors and does not necessarily represent the official views of the National Heart, Lung, and Blood Institute or the National Institutes of Health.

COPD Foundation Funding

The COPDGene® project is also supported by the COPD Foundation through contributions made to an Industry Advisory Board comprised of AstraZeneca, Boehringer Ingelheim, GlaxoSmithKline, Novartis, Pfizer, Siemens and Sunovion.

COPDGene® Investigators – Core Units

Administrative Center: James D. Crapo, MD (PI); Edwin K. Silverman, MD, PhD (PI); Barry J. Make, MD; Elizabeth A. Regan, MD, PhD

Genetic Analysis Center: Terri Beaty, PhD; Ferdouse Begum, PhD; Peter J. Castaldi, MD, MSc; Michael Cho, MD; Dawn L. DeMeo, MD, MPH; Adel R. Boueiz, MD; Marilyn G. Foreman, MD, MS; Eitan Halper-Stromberg; Lystra P. Hayden, MD, MMSc; Craig P. Hersh, MD, MPH; Jacqueline Hetmanski, MS, MPH; Brian D. Hobbs, MD; John E. Hokanson, MPH, PhD; Nan Laird, PhD; Christoph Lange, PhD; Sharon M. Lutz, PhD; Merry-Lynn McDonald, PhD; Margaret M. Parker, PhD; Dandi Qiao, PhD; Elizabeth A. Regan, MD, PhD; Edwin K. Silverman, MD, PhD; Emily S. Wan, MD; Sungho Won, Ph.D.; Phuwanat Sakornsakolpat, M.D.; Dmitry Prokopenko, Ph.D.

Imaging Center: Mustafa Al Qaisi, MD; Harvey O. Coxson, PhD; Teresa Gray; MeiLan K. Han, MD, MS; Eric A. Hoffman, PhD; Stephen Humphries, PhD; Francine L. Jacobson, MD, MPH; Philip F. Judy, PhD; Ella A. Kazerooni, MD; Alex Kluiber; David A. Lynch, MB; John D. Newell, Jr., MD; Elizabeth A. Regan, MD, PhD; James C. Ross, PhD; Raul San Jose Estepar, PhD; Joyce Schroeder, MD; Jered Sieren; Douglas Stinson; Berend C. Stoel, PhD; Juerg Tschirren, PhD; Edwin Van Beek, MD, PhD; Bram van Ginneken, PhD; Eva van Rikxoort, PhD; George Washko, MD; Carla G. Wilson, MS;

PFT QA Center, Salt Lake City, UT: Robert Jensen, PhD

Data Coordinating Center and Biostatistics, National Jewish Health, Denver, CO: Douglas Everett, PhD; Jim Crooks, PhD; Camille Moore, PhD; Matt Strand, PhD; Carla G. Wilson, MS

Epidemiology Core, University of Colorado Anschutz Medical Campus, Aurora, CO: John E. Hokanson, MPH, PhD; John Hughes, PhD; Gregory Kinney, MPH, PhD; Sharon M. Lutz, PhD; Katherine Pratte, MSPH; Kendra A. Young, PhD

Mortality Adjudication Core: Surya Bhatt, MD; Jessica Bon, MD; MeiLan K. Han, MD, MS; Barry Make, MD; Carlos Martinez, MD, MS; Susan Murray, ScD; Elizabeth Regan, MD; Xavier Soler, MD; Carla G. Wilson, MS

Biomarker Core: Russell P. Bowler, MD, PhD; Katerina Kechris, PhD; Farnoush Banaei-Kashani, Ph.D

COPDGene® Investigators – Clinical Centers

Ann Arbor VA: Jeffrey L. Curtis, MD; Carlos H. Martinez, MD, MPH; Perry G. Pernicano, MD

Baylor College of Medicine, Houston, TX: Nicola Hanania, MD, MS; Philip Alapat, MD; Mustafa Atik, MD; Venkata Bandi, MD; Aladin Boriek, PhD; Kalpatha Guntupalli, MD; Elizabeth Guy, MD; Arun Nachiappan, MD; Amit Parulekar, MD;

Brigham and Women's Hospital, Boston, MA: Dawn L. DeMeo, MD, MPH; Craig Hersh, MD, MPH; Francine L. Jacobson, MD, MPH; George Washko, MD

Columbia University, New York, NY: R. Graham Barr, MD, DrPH; John Austin, MD; Belinda D'Souza, MD; Gregory D.N. Pearson, MD; Anna Rozenshtein, MD, MPH, FACR; Byron Thomashow, MD

Duke University Medical Center, Durham, NC: Neil MacIntyre, Jr., MD; H. Page McAdams, MD; Lacey Washington, MD

HealthPartners Research Institute, Minneapolis, MN: Charlene McEvoy, MD, MPH; Joseph Tashjian, MD

Johns Hopkins University, Baltimore, MD: Robert Wise, MD; Robert Brown, MD; Nadia N. Hansel, MD, MPH; Karen Horton, MD; Allison Lambert, MD, MHS; Nirupama Putcha, MD, MHS

Los Angeles Biomedical Research Institute at Harbor UCLA Medical Center, Torrance, CA: Richard Casaburi, PhD, MD; Alessandra Adami, PhD; Matthew Budoff, MD; Hans Fischer, MD; Janos Porszasz, MD, PhD; Harry Rossiter, PhD; William Stringer, MD

Michael E. DeBakey VAMC, Houston, TX: Amir Sharafkhaneh, MD, PhD; Charlie Lan, DO

Minneapolis VA: Christine Wendt, MD; Brian Bell, MD

Morehouse School of Medicine, Atlanta, GA: Marilyn G. Foreman, MD, MS; Eugene Berkowitz, MD, PhD; Gloria Westney, MD, MS

National Jewish Health, Denver, CO: Russell Bowler, MD, PhD; David A. Lynch, MB

Reliant Medical Group, Worcester, MA: Richard Rosiello, MD; David Pace, MD

Temple University, Philadelphia, PA: Gerard Criner, MD; David Ciccolella, MD; Francis Cordova, MD; Chandra Dass, MD; Gilbert D'Alonzo, DO; Parag Desai, MD; Michael Jacobs, PharmD; Steven Kelsen, MD, PhD; Victor Kim, MD; A. James Mamary, MD; Nathaniel Marchetti, DO; Aditi Satti, MD; Kartik Shenoy, MD; Robert M. Steiner, MD; Alex Swift, MD; Irene Swift, MD; Maria Elena Vega-Sanchez, MD

University of Alabama, Birmingham, AL: Mark Dransfield, MD; William Bailey, MD; Surya Bhatt, MD; Anand Iyer, MD; Hrudaya Nath, MD; J. Michael Wells, MD

University of California, San Diego, CA: Joe Ramsdell, MD; Paul Friedman, MD; Xavier Soler, MD, PhD; Andrew Yen, MD

University of Iowa, Iowa City, IA: Alejandro P. Comellas, MD; Karin F. Hoth, PhD; John Newell, Jr., MD; Brad Thompson, MD

University of Michigan, Ann Arbor, MI: MeiLan K. Han, MD, MS; Ella Kazerooni, MD; Carlos H. Martinez, MD, MPH

University of Minnesota, Minneapolis, MN: Joanne Billings, MD; Abbie Begnaud, MD; Tadashi Allen, MD

University of Pittsburgh, Pittsburgh, PA: Frank Sciurba, MD; Jessica Bon, MD; Divay Chandra, MD, MSc; Carl Fuhrman, MD; Joel Weissfeld, MD, MPH

University of Texas Health Science Center at San Antonio, San Antonio, TX: Antonio Anzueto, MD; Sandra Adams, MD; Diego Maselli-Caceres, MD; Mario E. Ruiz, MD

The **ECLIPSE** study (NCT00292552; GSK code SCO104960) was funded by GlaxoSmithKline.

ECLIPSE Investigators — *Bulgaria:* Y. Ivanov, Pleven; K. Kostov, Sofia. *Canada:* J. Bourbeau, Montreal; M. Fitzgerald, Vancouver, BC; P. Hernandez, Halifax, NS; K. Killian, Hamilton, ON; R. Levy, Vancouver, BC; F. Maltais, Montreal; D. O'Donnell, Kingston, ON. *Czech Republic:* J. Krepelka, Prague. *Denmark:* J. Vestbo, Hvidovre. *The Netherlands:*E. Wouters, Horn-Maastricht. *New Zealand:* D. Quinn, Wellington. *Norway:* P. Bakke, Bergen. *Slovenia:* M. Kosnik, Golnik. *Spain:* A. Agusti, J. Sauleda, P. de Mallorca. *Ukraine:*Y. Feschenko, V. Gavrisyuk, L. Yashina, Kiev; N. Monogarova, Donetsk. *United Kingdom:* P. Calverley, Liverpool; D. Lomas, Cambridge; W. MacNee, Edinburgh; D. Singh, Manchester; J. Wedzicha, London. *United States:* A. Anzueto, San Antonio, TX; S. Braman, Providence, RI; R. Casaburi, Torrance CA; B. Celli, Boston; G. Giessel, Richmond, VA; M. Gotfried, Phoenix, AZ; G. Greenwald, Rancho Mirage, CA; N. Hanania, Houston; D. Mahler, Lebanon, NH; B. Make, Denver; S. Rennard, Omaha, NE; C. Rochester, New Haven, CT; P. Scanlon, Rochester, MN; D. Schuller, Omaha, NE; F. Sciurba, Pittsburgh; A. Sharafkhaneh, Houston; T. Siler, St. Charles, MO; E. Silverman, Boston; A. Wanner, Miami; R. Wise, Baltimore; R. ZuWallack, Hartford, CT.

ECLIPSE Steering Committee: H. Coxson (Canada), C. Crim (GlaxoSmithKline, USA), L. Edwards (GlaxoSmithKline, USA), D. Lomas (UK), W. MacNee (UK), E. Silverman (USA), R. Tal Singer (Co-chair, GlaxoSmithKline, USA), J. Vestbo (Co-chair, Denmark), J. Yates (GlaxoSmithKline, USA).

ECLIPSE Scientific Committee: A. Agusti (Spain), P. Calverley (UK), B. Celli (USA), C. Crim (GlaxoSmithKline, USA), B. Miller (GlaxoSmithKline, USA), W. MacNee (Chair, UK), S. Rennard (USA), R. Tal-Singer (GlaxoSmithKline, USA), E. Wouters (The Netherlands), J. Yates (GlaxoSmithKline, USA).

MESA

MESA and the MESA SHARe project are conducted and supported by the National Heart, Lung, and Blood Institute (NHLBI) in collaboration with MESA investigators. The MESA Lung Study is supported by R01-HL077612 and R01-HL093081. Support for

MESA is provided by contracts HHSN268201500003I, N01-HC-95159, N01-HC-95160, N01-HC-95161, N01-HC-95162, N01-HC-95163, N01-HC-95164, N01-HC-95165, N01-HC-95166, N01-HC-95167, N01-HC-95168, N01-HC-95169, UL1-TR-000040, UL1-TR-001079, UL1-TR-001420. MESA Family is conducted and supported by the National Heart, Lung, and Blood Institute (NHLBI) in collaboration with MESA investigators. Support is provided by grants and contracts R01HL071051, R01HL071205, R01HL071250, R01HL071251, R01HL071258, R01HL071259, and by the National Center for Research Resources, Grant UL1RR033176. The provision of genotyping data was supported in part by the National Center for Advancing Translational Sciences, CTSI grant UL1TR001881, and the National Institute of Diabetes and Digestive and Kidney Disease Diabetes Research Center (DRC) grant DK063491 to the Southern California Diabetes Endocrinology Research Center. Funding for SHARe genotyping was provided by NHLBI Contract N02-HL-64278.

CHS

This CHS research was supported by NHLBI contracts HHSN268201200036C, HHSN268200800007C, HHSN268201800001C, N01HC55222, N01HC85079, N01HC85080, N01HC85081, N01HC85082, N01HC85083, N01HC85086; and NHLBI grants U01HL080295, R01HL087652, R01HL105756, R01HL103612, R01HL120393, and U01HL130114 with additional contribution from the National Institute of Neurological Disorders and Stroke (NINDS). Additional support was provided through R01AG023629 from the National Institute on Aging (NIA). A full list of principal CHS investigators and institutions can be found at CHS-NHLBI.org.

The provision of genotyping data was supported in part by the National Center for Advancing Translational Sciences, CTSI grant UL1TR001881, and the National Institute of Diabetes and Digestive and Kidney Disease Diabetes Research Center (DRC) grant DK063491 to the Southern California Diabetes Endocrinology Research Center.

Rotterdam Study

The Rotterdam Study is supported by the Erasmus MC and Erasmus University Rotterdam; the Netherlands Organisation for Scientific Research (NWO); the Netherlands Organisation for Health Research and Development (ZonMW); the Research Institute for Diseases in the Elderly (RIDE); the Netherlands Genomics Initiative; the Ministry of Education, Culture and Science; the Ministry of Health, Welfare and Sports; the European Commission (DG XII); and the Municipality of Rotterdam.

The funding source had no involvement in the collection, analysis, writing, interpretation, or in the decision to submit the paper for publication.

SPIROMICS
The Subpopulations and Intermediate Outcomes in COPD Study (SPIROMICS) was supported by contracts from the NIH/NHLBI (HHSN268200900013C, HHSN268200900014C, HHSN268200900015C, HHSN268200900016C, HHSN268200900017C, HHSN268200900018C, HHSN268200900019C, HHSN268200900020C), which were supplemented by contributions made through the Foundation for the NIH from AstraZeneca; Bellerophon Therapeutics; Boehringer-Ingelheim Pharmaceuticals, Inc; Chiesi Farmaceutici SpA; Forest Research Institute, Inc; GSK; Grifols Therapeutics, Inc; Ikaria, Inc; Nycomed GmbH; Takeda Pharmaceutical Company; Novartis Pharmaceuticals Corporation; Regeneron Pharmaceuticals, Inc; and Sanofi.

UK Biobank
The UK Biobank analysis was conducted under approved UK Biobank data application number 648.

Supplementary Methods

Derivation of polygenic risk scores

The PRS was penalized using lasso regression. Lasso regression is a form of L1 penalized regression where absolute values of coefficients that are closer to zero are more strongly penalized. As a result, certain coefficients can be shrunk to zero, which provides for a simpler model. The lassosum R package extends this concept. The shrinkage parameter is estimated from the covariance matrix, and the lambda is estimated from the PRS that most strongly correlates with the phenotype in the validation set (i.e. GenKOLS in this study). In addition, this takes into account linkage disequilibrium with a moderate degree of clumping and thresholding.[23]

A clinical risk score (CRS) was derived in UK Biobank. We included participants with ≥ 10 pack-years of smoking history, as this is similar exposure as the COPDGene and ECLIPSE testing cohorts. We then constructed a logistic regression model in which the outcome was moderate-to-severe COPD and the predictors included age, sex, and pack-years of cigarette smoking. This model was used to calculate CRSs in COPDGene and ECLIPSE.

Supplementary Tables

Table S1: Association of PRS with frequent (> 1 exacerbation in 12 months) and severe exacerbations (exacerbation requiring emergency room visit or hospitalization). Logistic regression models were adjusted for age, sex, pack years of smoking, ± lung function (FEV_1 and FEV_1/FVC).

Outcome	Model	*COPDGene*		*ECLIPSE*	
		OR (95% CI)	*P*	*OR (95% CI)*	*P*
Severe Exacerbations	Adj. (age, sex, pack years)	1.26 (1.12 - 1.42)	0.00011	1.12 (1.01 - 1.23)	0.027
	Adj. (age, sex, pack years, lung function)	0.99 (0.87 - 1.13)	0.89	1.01 (0.9 - 1.12)	0.92
Frequent Exacerbations	Adj. (age, sex, pack years)	1.04 (1.02 - 1.05)	6.80E-07	1.03 (1.01 - 1.05)	0.015
	Adj. (age, sex, pack years, lung function)	1 (0.98 - 1.01)	0.5	1 (0.97 - 1.02)	0.66

Table S2: Meta-analysis of AUCs in European cohorts comparing models with only combined PRS, only clinical risk factors, and clinical risk factors with combined PRS. The model with both clinical risk factors and PRS had a significantly higher AUC than the model with clinical risk factors alone ($p = 1.6e-39$). AUCs were meta-analyzed by two methods: (1) inverse variance weighting, and (2) effective sample size weighting.

Model	*AUC weighted by inverse variance (95% CI)*	*AUC weighted by sample size (95% CI)*
Combined polygenic score (PRS)	0.67 (0.663-0.681)	0.66 (0.635-0.687)
Risk factors (age, sex, and smoking pack-years)	0.76 (0.748-0.763)	0.73 (0.706-0.752)
Risk factors and PRS	0.80 (0.791-0.806)	0.77 (0.75-0.793)

Table S3: AUC, confidence intervals, and p-values for each model and comparison in Figure 4.

Model	*Cohort*	*AUC*	*AUC lower 95% CI*	*AUC upper 95% CI*	*P (risk factors vs PRS)*	*P (PRS+risk factors vs. risk factors)*
Combined polygenic score (PRS)	COPDGene NHW	0.689	0.673	0.704	NA	NA
Risk factors (age, sex, and smoking pack-years)	COPDGene NHW	0.764	0.75	0.778	2.69E-13	2.70E-25
Risk factors and PRS	COPDGene NHW	0.811	0.798	0.823	NA	NA
Combined polygenic score (PRS)	ECLIPSE	0.713	0.671	0.756	NA	NA
Risk factors (age, sex, and smoking pack-years)	ECLIPSE	0.824	0.788	0.86	5.41E-05	0.0042
Risk factors and PRS	ECLIPSE	0.852	0.818	0.887	NA	NA
Combined polygenic score (PRS)	NETT/NAS	0.745	0.711	0.779	NA	NA
Risk factors (age, sex, and smoking pack-years)	NETT/NAS	0.899	0.879	0.92	9.12E-19	0.0104
Risk factors and PRS	NETT/NAS	0.912	0.893	0.931	NA	NA
Risk factors (age, sex, and smoking pack-years)	CHS EA	0.758	0.734	0.781	1.63E-06	6.66E-06
Combined polygenic score (PRS)	CHS EA	0.669	0.643	0.695	NA	NA
Risk factors and PRS	CHS EA	0.798	0.777	0.82	NA	NA
Risk factors (age, sex, and smoking pack-years)	MESA NHW	0.741	0.703	0.779	0.0215	0.000603
Combined polygenic score (PRS)	MESA NHW	0.673	0.632	0.714	NA	NA
Risk factors and PRS	MESA NHW	0.794	0.76	0.828	NA	NA
Risk factors (age, sex, and smoking pack-years)	LHS	0.576	0.553	0.598	0.542	3.47E-05
Combined polygenic score (PRS)	LHS	0.585	0.563	0.608	NA	NA
Risk factors and PRS	LHS	0.617	0.594	0.639	NA	NA
Risk factors (age, sex, and smoking pack-years)	SPIROMICS NHW	0.666	0.637	0.694	0.425	3.16E-09
Combined polygenic score (PRS)	SPIROMICS NHW	0.682	0.654	0.71	NA	NA

Risk factors and PRS	SPIROMICS NHW	0.742	0.716	0.768	NA	NA
Risk factors (age, sex, and smoking pack-years)	RS1	0.716	0.67	0.763	0.494	0.0013
Combined polygenic score (PRS)	RS1	0.692	0.642	0.741	NA	NA
Risk factors and PRS	RS1	0.781	0.74	0.823	NA	NA
Risk factors (age, sex, and smoking pack-years)	RS2	0.79	0.742	0.838	2.14E-05	0.115
Combined polygenic score (PRS)	RS2	0.628	0.568	0.687	NA	NA
Risk factors and PRS	RS2	0.808	0.763	0.854	NA	NA
Risk factors (age, sex, and smoking pack-years)	RS3	0.804	0.766	0.842	0.00118	0.0662
Combined polygenic score (PRS)	RS3	0.7	0.652	0.749	NA	NA
Risk factors and PRS	RS3	0.833	0.796	0.869	NA	NA

Table S4: Performance characteristics of a clinical risk score (CRS) trained in UK Biobank participants with ≥ 10 pack-years of smoking history, the PRS, and both the CRS and PRS combined. The threshold was chosen by optimal Youden index. “PPV” = positive predictive value. “NPV” = negative predictive value.

Score	*Threshold*	*Accuracy*	*PPV*	*NPV*	*Sensitivity*	*Specificity*
CRS	0.55	0.6	0.62	0.52	0.85	0.23
PRS	0.5	0.65	0.67	0.61	0.82	0.4
CRS + PRS	0.49	0.73	0.75	0.7	0.82	0.61

Table S5: The association of the PRS with % LAA < -950 HU and WAP after adjusting for baseline FEV_1 % predicted.

Score	*trait*	*Beta*	*Se*	*p*
PRS	% LAA < -950 HU	-0.03	0.017	0.08
PRS	WAP	0.37	0.042	4.1E-18

Table S6: Results of ordinal logistic regression showing association of the combined polygenic risk score with visual emphysema severity category. [24]

Predictor	OR (95% CI)	p-value
Combined PRS	1.2 (1.13 - 1.27)	2.70E-10
Age	1.04 (1.03 - 1.04)	9.10E-27
Gender	1.06 (0.95 - 1.19)	0.3
Pack-years	1.03 (1.02 - 1.03)	5.00E-89
Principal component 1	0 (0 - 0)	1.90E-05
0|1	18.02 (11.36 - 28.59)	1.10E-34
1|2	39.72 (24.92 - 63.29)	4.20E-54
2|3	102.17 (63.61 - 164.12)	1.30E-81
3|4	297.53 (183.35 - 482.83)	1.20E-117
4|5	1397.13 (839.12 - 2326.22)	1.30E-170

Supplementary Figures

Figure S1: Flow diagram of participants included in this analysis

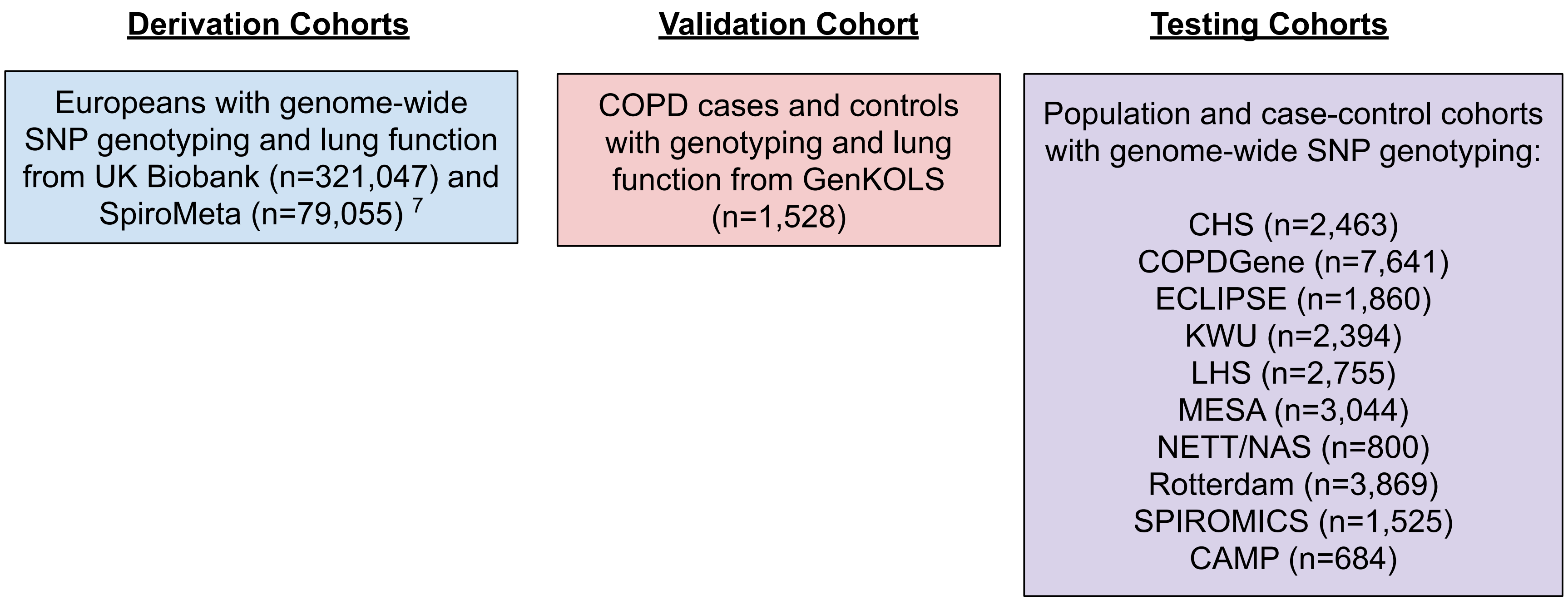

Figure S2: Flow diagram of variants included in the polygenic risk scores

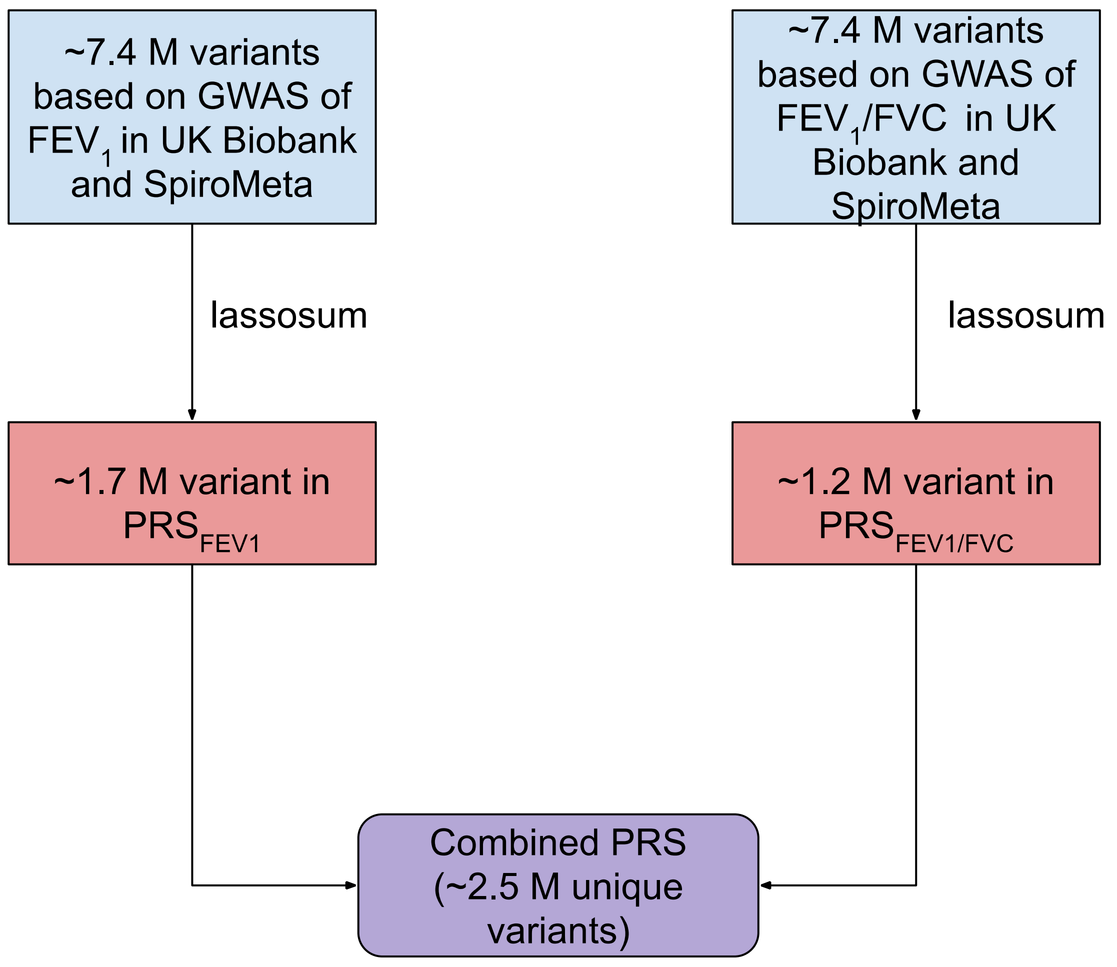

Figure S3: Plot showing effect sizes of genetic loci associated with FEV_1/FVC in meta-analysis of UK Biobank and SpiroMeta.

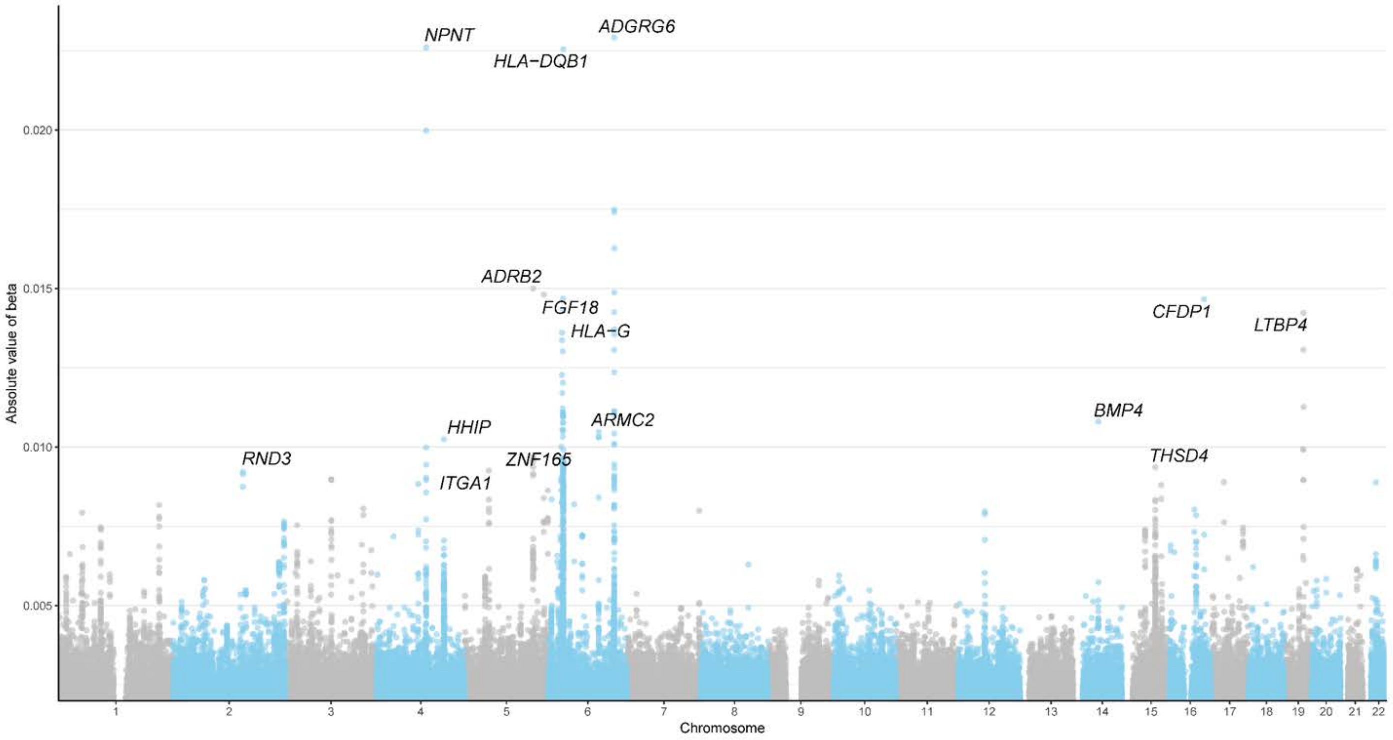

Figure S4: Area-under-the-curve (AUC) measures to predict COPD for PRSs validated in one cohort and tested in others. "PRSfev1" indicates a PRS using FEV1, "PRSratio" using ratio, and "PRSfev1AndRatio" the combined score used as the main analysis in the manuscript. The suffixes indicate the cohort used for validation. "ASP" indicates that the model was adjusted for age, sex, and pack-years of smoking. The AUC for measures are the measures calculated in testing cohorts, labeled on the x-axis. "cgNhw" = COPDGene NHW, "cgAa" = COPDGene AA.

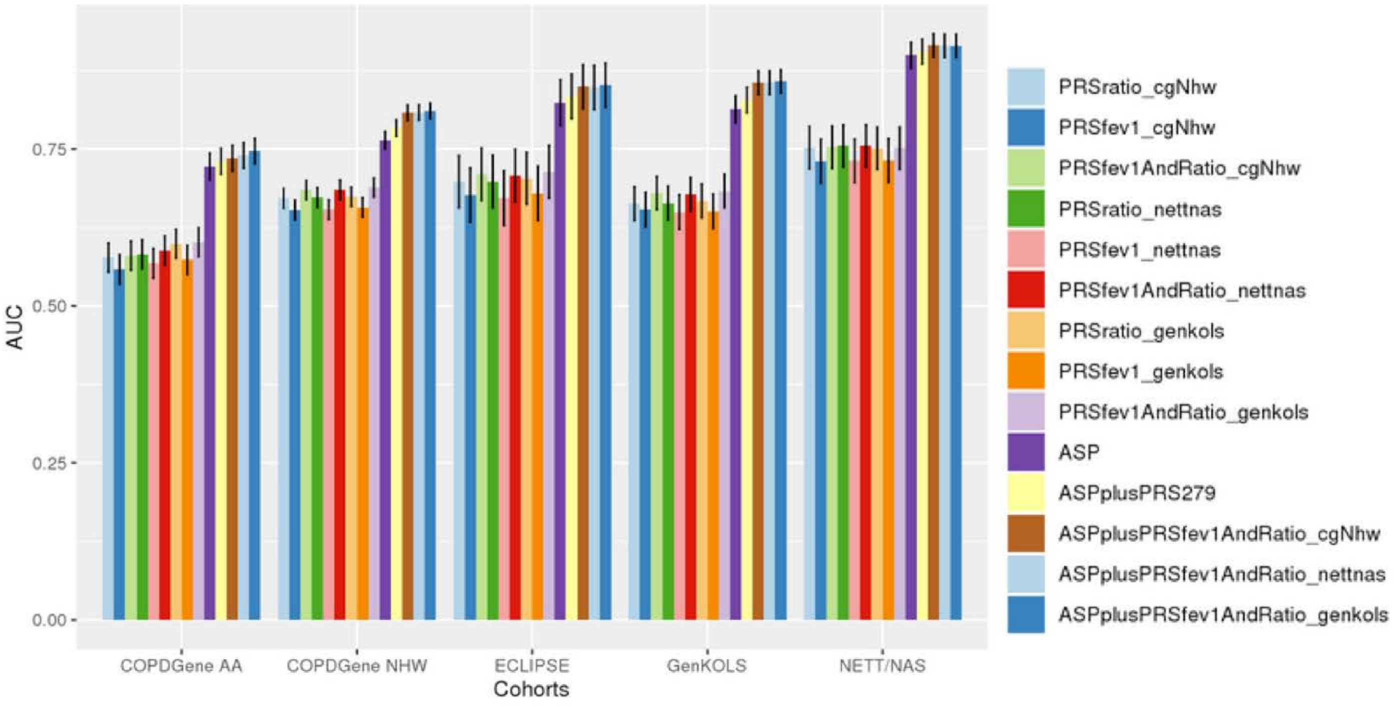

Figure S5: Representative scatterplot showing minimal correlation between the combined FEV_1 and FEV_1/FVC PRS in the COPDGene NHW population with smoking intensity. Similar scatterplots were observed in COPDGene AA, GenKOLS, ECLIPSE, and NETT/NAS.

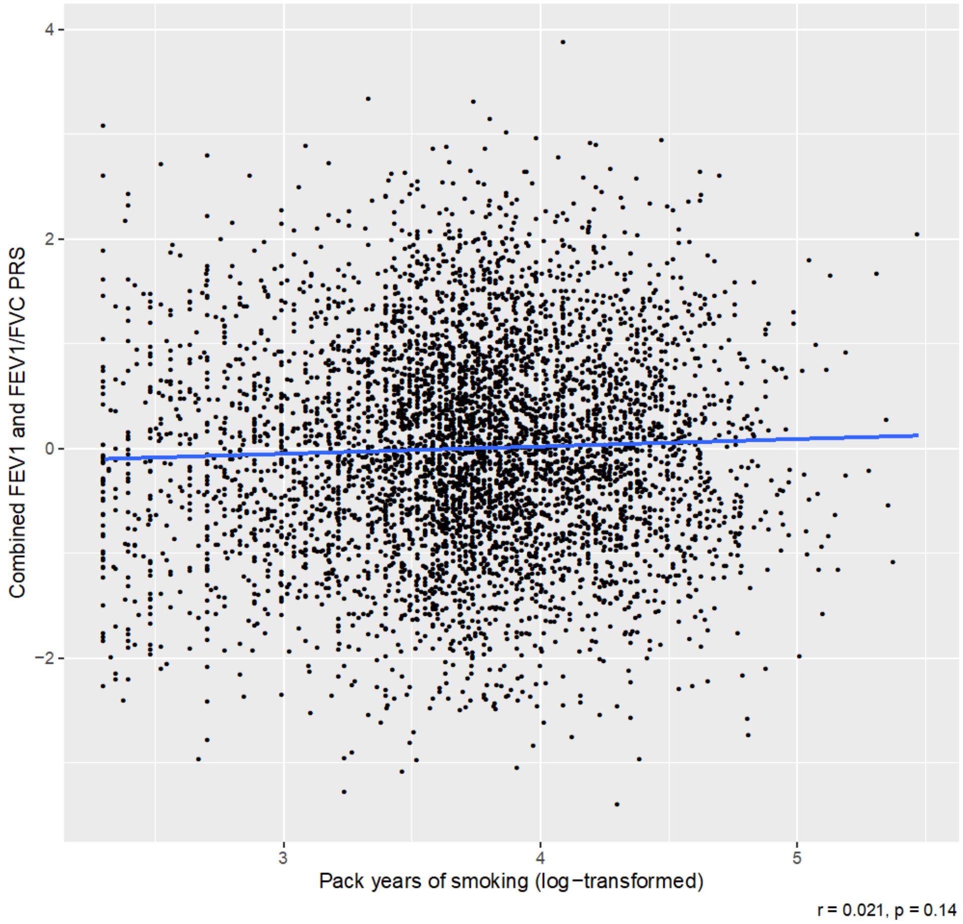

Figure S6: Funnel plot of the combined FEV_1 and FEV_1/FVC PRS effect sizes versus standard errors for each cohort.

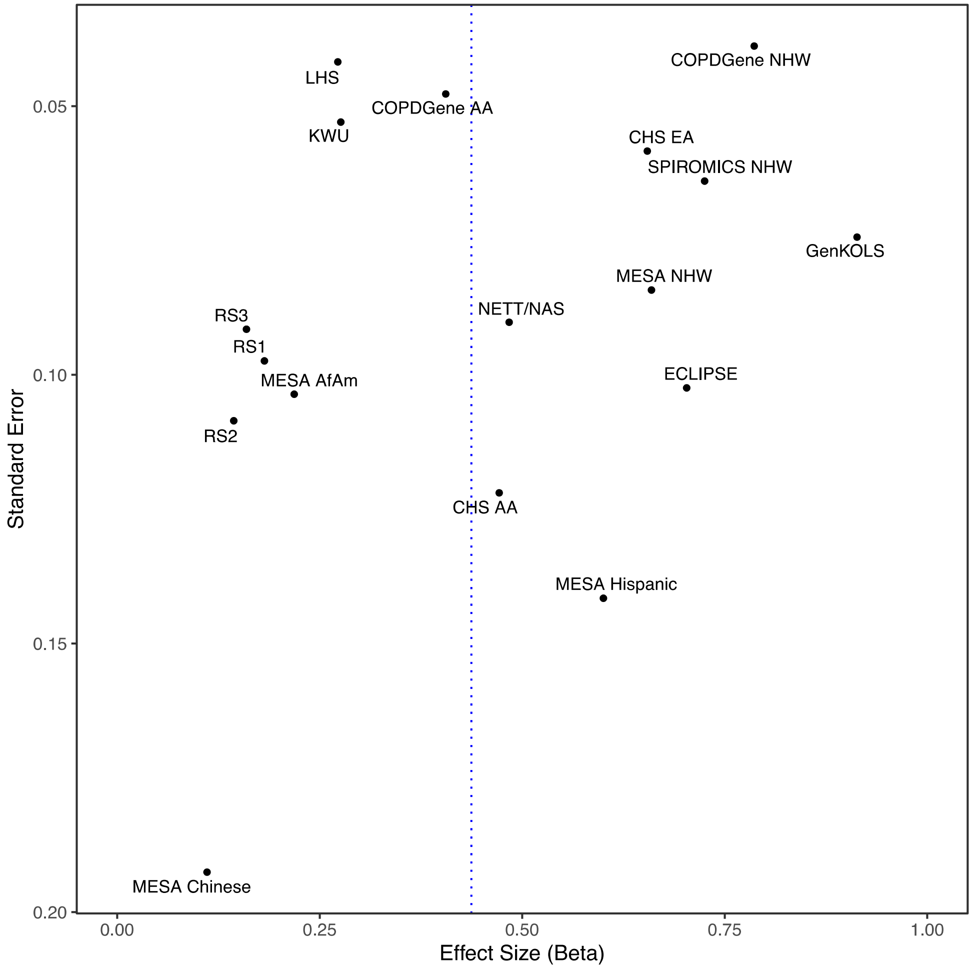

Figure S7: Correlation of FEV1 and FEV1/FVC PRSs with GOLD spirometry grades in COPDGene NHW population. PRISm = preserved ratio with impaired spirometry.

A)

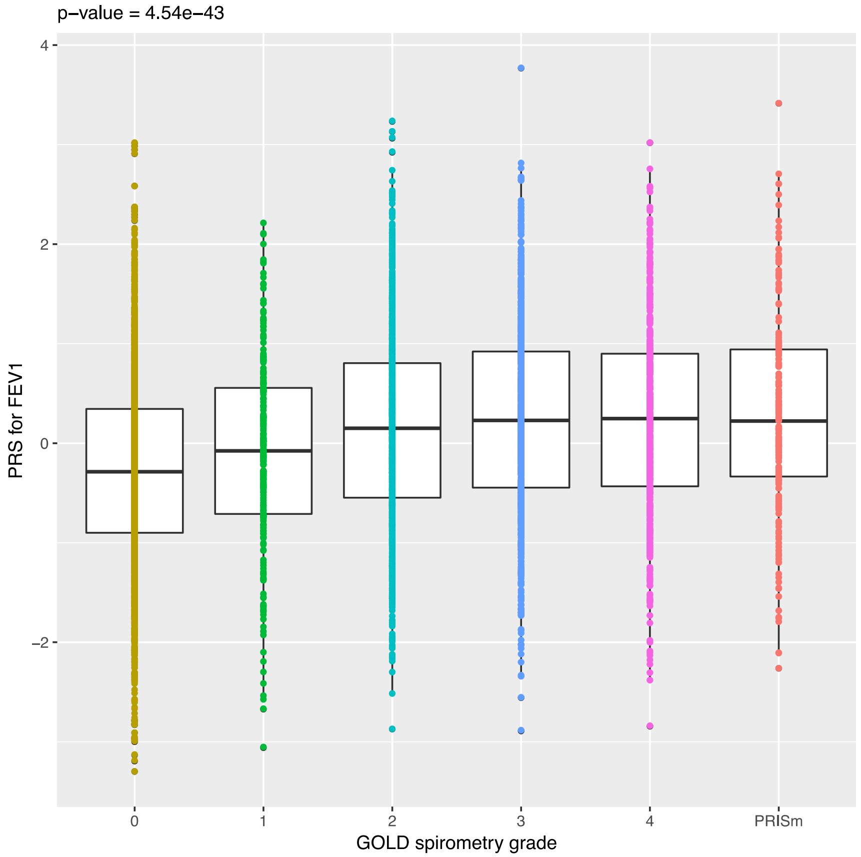

B)

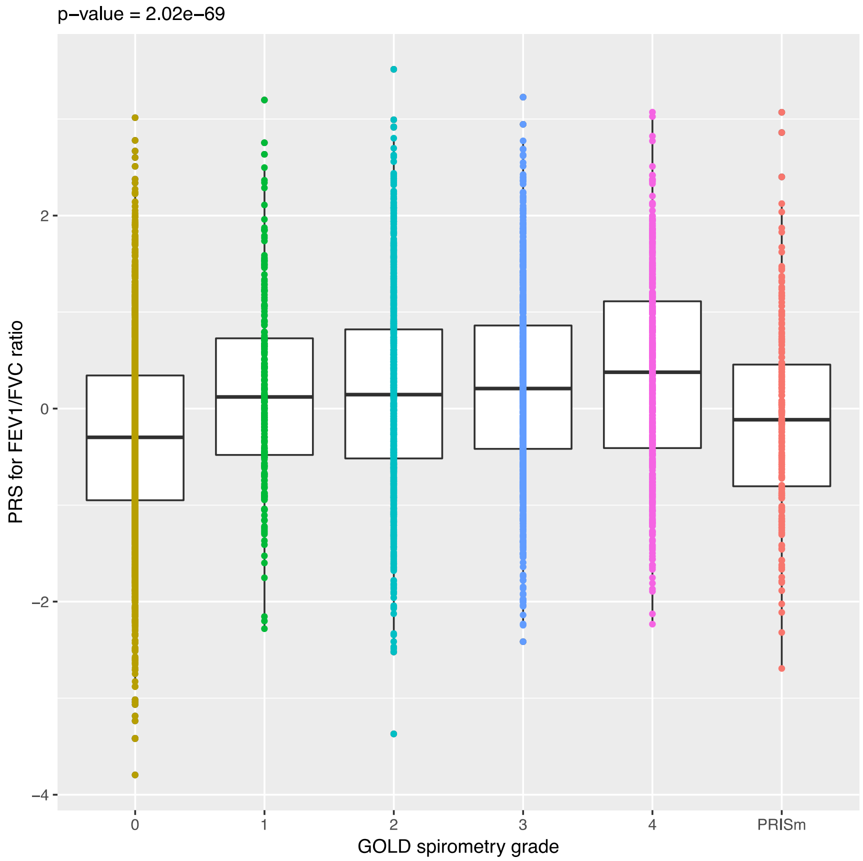
p−value = 2.02e−69
PRS for FEV1/FVC ratio
2
0
−2
−4
0
1
2
3
4
PRISm
GOLD spirometry grade

Figure S8: Scatterplot of controls and COPD cases in the COPDGene NHW cohort based on pack-years of smoking and polygenic risk based on PRS.

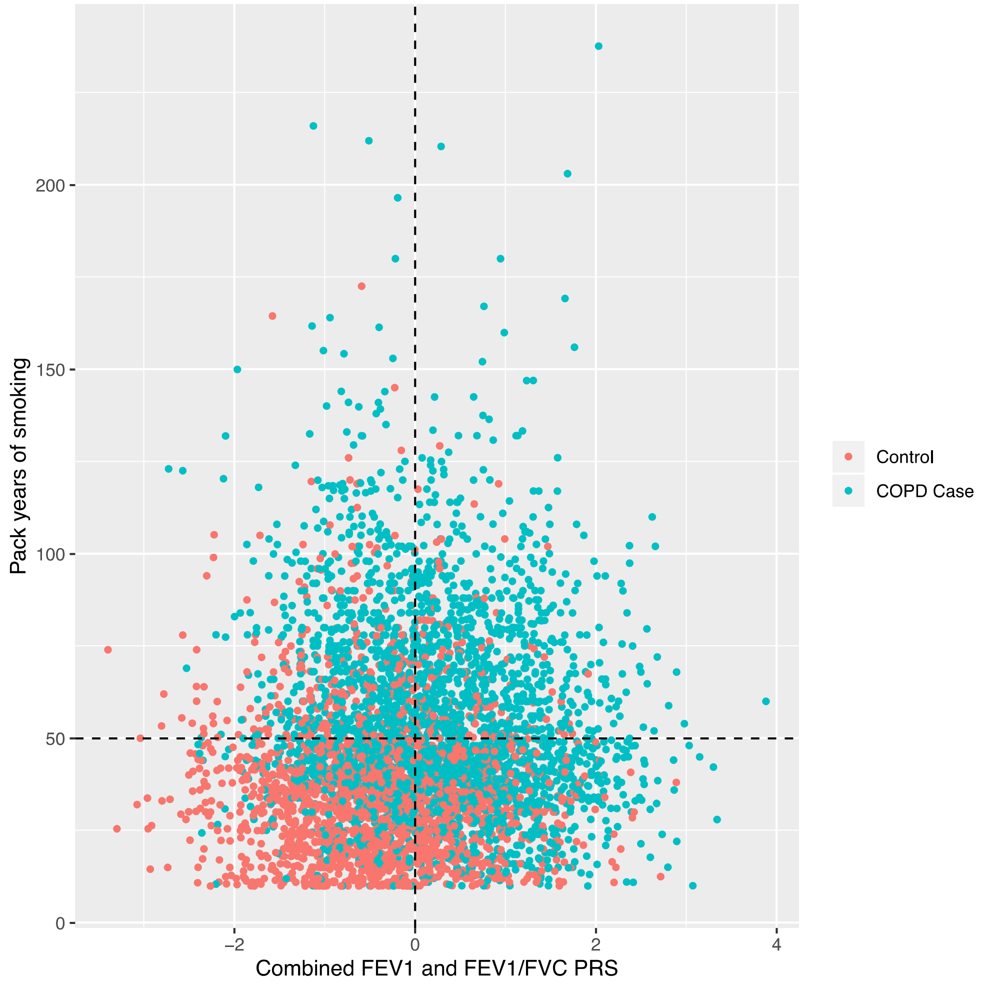

Figure S9: Comparison of AUCs for each of three PRSs across all cohorts.

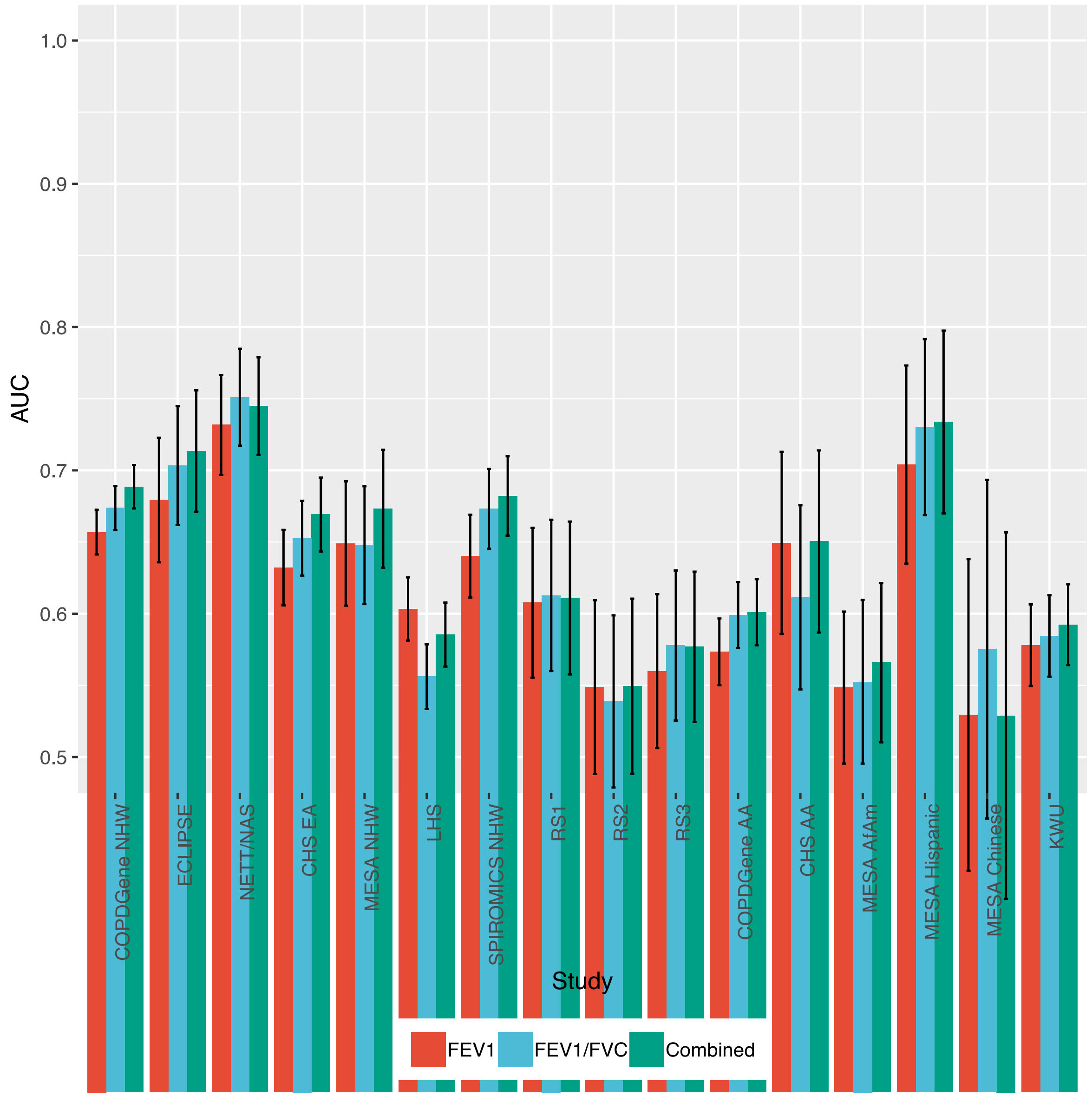

Figure S10: Receiver operator characteristic (ROC) curves for a clinical risk score (CRS) trained in UK Biobank participants with ≥ 10 pack years of cigarette smoking, the PRS, and both the CRS and PRS together, tested in the COPDGene and ECLIPSE cohorts. COPDGene p-value (AUC PRS vs CRS) = 1.1e-36; ECLIPSE p-value (AUC PRS vs CRS) = 3.0e-5. COPDGene p-value (AUC PRS+CRS vs PRS) = 3.9e-64; ECLIPSE (AUC PRS+CRS vs PRS) = 5.8e-11.

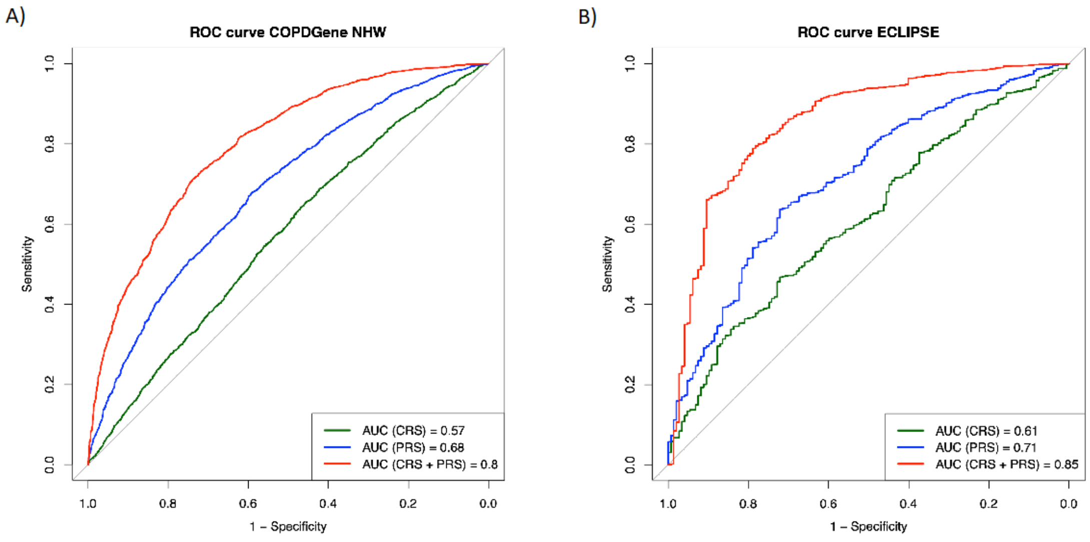

Figure S11: Association of PRS with CT imaging phenotypes. Meta-analysis beta coefficients are shown. “*” indicates that p-value is below Bonferroni-corrected significance level of 0.0025 (0.05/20 imaging comparisons).

A: %LAA < -950 HU (n=9,340); CI (Bonferroni-adjusted): 0.1-0.17; p = 2.3e-26*

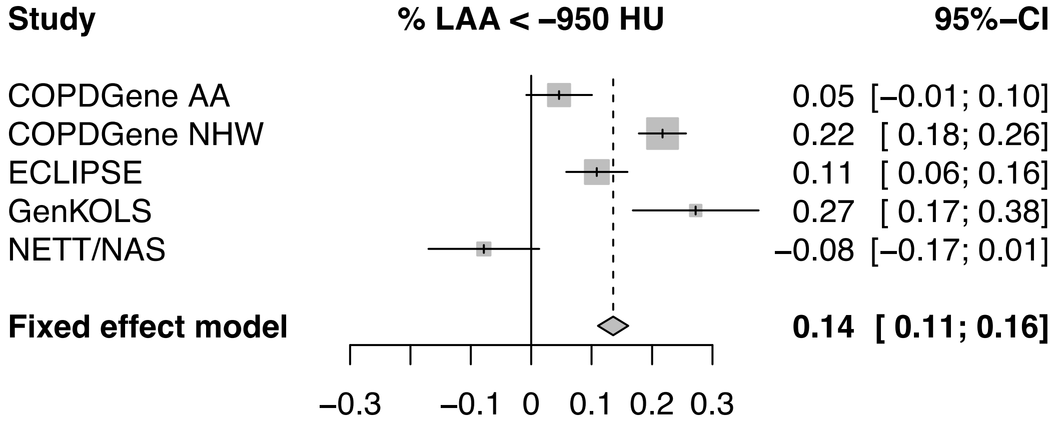

B: Perc15 (n=9,340); CI (Bonferroni-adjusted): -3.5--2.0; p = 1.1e-25*

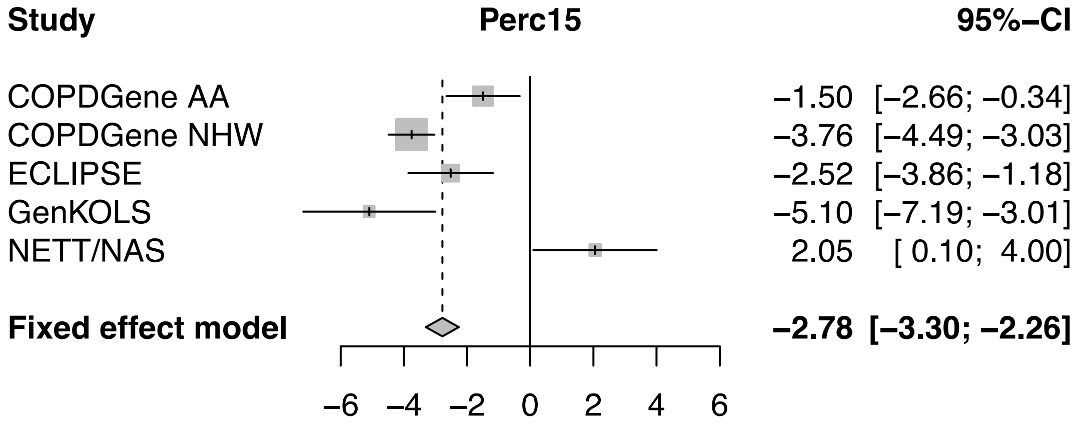

C: Pi10 (n=8,820); CI (Bonferroni-adjusted): 0.0074-0.015; p = 8.8e-16*

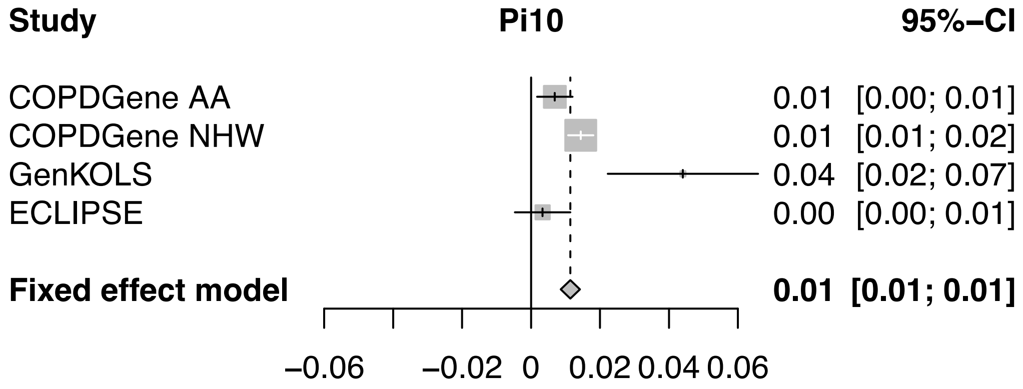

D: WAP (n=9,310); CI (Bonferroni-adjusted): 0.59-0.77; p = 1.4e-95*

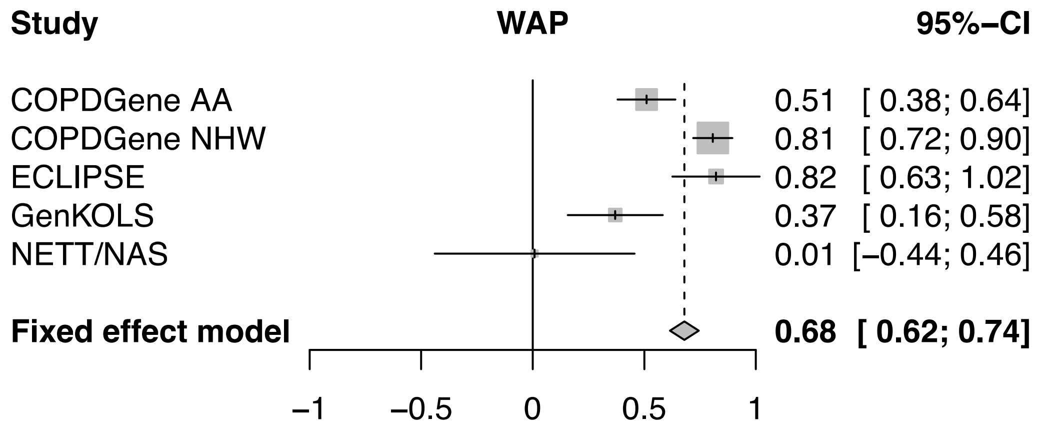

E: Gas trapping (n=6,110); CI (Bonferroni-adjusted): 0.12-0.19; p = 6.1e-39*

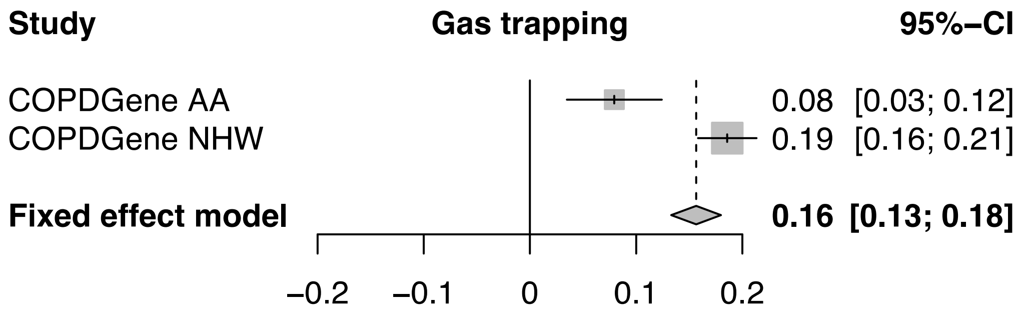

F: Qualitative emphysema (n=2,600); CI (Bonferroni-adjusted): -0.03-0.13; p=0.08

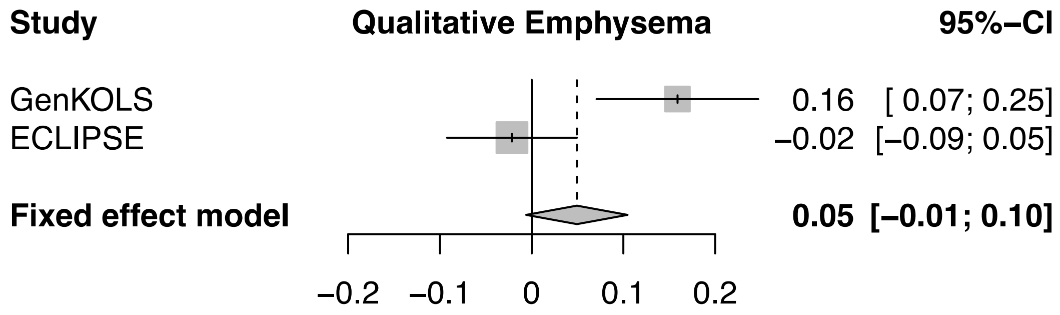

Figure S12: Odds ratio for the combined polygenic risk score and the association with reduced compared to normal lung function growth curves in the CAMP study.

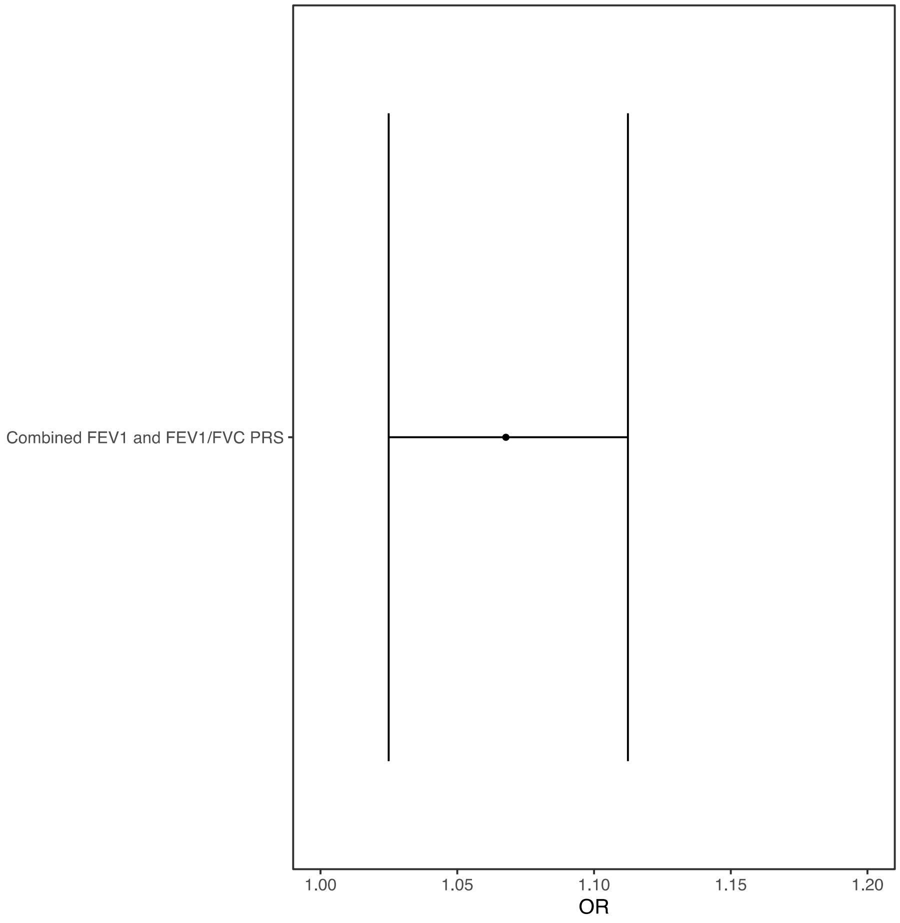

References

1 Regan EA, Hokanson JE, Murphy JR, *et al.* Genetic Epidemiology of COPD (COPDGene) Study Design. *COPD J Chronic Obstr Pulm Dis* 2011; **7**: 32–43.

2 McCarthy S, Das S, Kretzschmar W, *et al.* A reference panel of 64,976 haplotypes for genotype imputation. *Nat Genet* 2016; **48**: 1279–83.

3 Zhu G, Warren L, Aponte J, *et al.* The SERPINE2 gene is associated with chronic obstructive pulmonary disease in two large populations. *Am J Respir Crit Care Med* 2007; **176**: 167–73.

4 Patel BD, Coxson HO, Pillai SG, *et al.* Airway wall thickening and emphysema show independent familial aggregation in chronic obstructive pulmonary disease. *Am J Respir Crit Care Med* 2008; **178**: 500–5.

5 Fishman A, Martinez F, Naunheim K, *et al.* A randomized trial comparing lung-volume-reduction surgery with medical therapy for severe emphysema. *N Engl J Med* 2003; **348**: 2059–73.

6 Pillai SG, Ge D, Zhu G, *et al.* A genome-wide association study in chronic obstructive pulmonary disease (COPD): identification of two major susceptibility loci. *PLoS Genet* 2009; **5**: e1000421.

7 Bell B, Rose CL, Damon D. The Normative Aging Study: an interdisciplinary and longitudinal study of health and aging. *Aging Hum Dev* 1972; **3**: 5–17.

8 Cho MH, Boutaoui N, Klanderman BJ, *et al.* Variants in FAM13A are associated with chronic obstructive pulmonary disease. *Nat Genet* 2010; **42**: 200–2.

9 Cho MH, Castaldi PJ, Wan ES, *et al.* A genome-wide association study of COPD identifies a susceptibility locus on chromosome 19q13. *Hum Mol Genet* 2012; **21**: 947–57.

10 Vestbo J, Anderson W, Coxson HO, *et al.* Evaluation of COPD Longitudinally to Identify Predictive Surrogate End-points (ECLIPSE). *Eur Respir J* 2008; **31**: 869–73.

11 Hankinson JL, Kawut SM, Shahar E, Smith LJ, Stukovsky KH, Barr RG. Performance of American Thoracic Society-recommended spirometry reference values in a multiethnic sample of adults: the multi-ethnic study of atherosclerosis (MESA) lung study. *Chest* 2010; **137**: 138–45.

12 Sakornsakolpat P, Prokopenko D, Lamontagne M, *et al.* Genetic landscape of chronic obstructive pulmonary disease identifies heterogeneous cell-type and phenotype associations. *Nat Genet* 2019; **51**: 494–505.

13 Fried LP, Borhani NO, Enright P, *et al.* The Cardiovascular Health Study: design and rationale. *Ann Epidemiol* 1991; **1**: 263–

76.
14 Hofman A, Darwish Murad S, van Duijn CM, *et al.* The Rotterdam Study: 2014 objectives and design update. *Eur J Epidemiol* 2013; **28**: 889–926.
15 Couper D, LaVange LM, Han M, *et al.* Design of the Subpopulations and Intermediate Outcomes in COPD Study (SPIROMICS). *Thorax* 2014; **69**: 491–4.
16 Moon S, Kim YJ, Han S, *et al.* The Korea Biobank Array: Design and Identification of Coding Variants Associated with Blood Biochemical Traits. *Sci Rep* 2019; **9**: 1382.
17 Loh P-R, Danecek P, Palamara PF, *et al.* Reference-based phasing using the Haplotype Reference Consortium panel. *Nat Genet* 2016; **48**: 1443–8.
18 Das S, Forer L, Schönherr S, *et al.* Next-generation genotype imputation service and methods. *Nat Genet* 2016; **48**: 1284–7.
19 Kanner RE, Connett JE, Williams DE, Buist AS. Effects of randomized assignment to a smoking cessation intervention and changes in smoking habits on respiratory symptoms in smokers with early chronic obstructive pulmonary disease: the Lung Health Study. *Am J Med* 1999; **106**: 410–6.
20 Buist AS. The US Lung Health Study. *Respirology* 1997; **2**: 303–7.
21 McGeachie MJ, Yates KP, Zhou X, *et al.* Patterns of Growth and Decline in Lung Function in Persistent Childhood Asthma. *N Engl J Med* 2016; **374**: 1842–52.
22 Cristen J Willer 1, Serena Sanna, Anne U Jackson, Angelo Scuteri, Lori L Bonnycastle, Robert Clarke, Simon C Heath, Nicholas J Timpson, Samer S Najjar, Heather M Stringham, James Strait, William L Duren, Andrea Maschio, Fabio Busonero, Antonella Mulas, Gi GRA. Newly Identified Loci That Influence Lipid Concentrations and Risk of Coronary Artery Disease. *Nat Genet* 2008; **40**: 161–9.
23 Mak TSH, Porsch RM, Choi SW, Zhou X, Sham PC. Polygenic scores via penalized regression on summary statistics. *Genet Epidemiol* 2017; **41**: 469–80.
24 Lynch DA, Moore CM, Wilson C, *et al.* CT-based Visual Classification of Emphysema: Association with Mortality in the COPDGene Study. *Radiology* 2018; **288**: 859–66.
